# Supplementary material for: Chemical modification of uridine modulates mRNA-mediated proinflammatory and antiviral response in primary human macrophages
Source: Mol Ther Nucleic Acids. 2022 Jan 10;27:854–69. doi: 10.1016/j.omtn.2022.01.004 (PMC8807976; doi:10.1016/j.omtn.2022.01.004)
Supplement: Document S1. Figures S1–S9 and Table S1 [file mmc1.pdf]

OMTN, Volume *27*

Supplemental information

Chemical modification of uridine modulates mRNA-mediated proinflammatory and antiviral response in primary human macrophages

Hanieh Moradian, Toralf Roch, Larissa Anthofer, Andreas Lendlein, and Manfred Gossen

Supporting Information for

Chemical modification of uridine modulates mRNA-mediated proinflammatory and antiviral response in primary human macrophages

Hanieh Moradian[1, 2, 3], Toralf Roch[4, 5,6], Larissa Anthofer[1, 2], Andreas Lendlein[1, 2, 3], Manfred Gossen[1, 2, 1]

1 Institute of Active Polymers, Helmholtz-Zentrum Hereon, Kantstr. 55, 14513 Teltow, Germany
2 Berlin-Brandenburg Center for Regenerative Therapies (BCRT), Föhrerstr. 15, 13353 Berlin, Germany
3 Institute of Biochemistry and Biology, University of Potsdam, Karl-Liebknecht-Str. 24-25, 14476 Potsdam, Germany
4 Berlin Institute of Health at Charité – Universitätsmedizin Berlin, BIH Center for Regenerative Therapies (BCRT), Charitéplatz 1, 10117 Berlin, Germany
5 Charité – Universitätsmedizin Berlin, Corporate Member of Freie Universität Berlin, Humboldt-Universität zu Berlin, Berlin Center for Advanced Therapies, Augustenburger Platz 1, 13353 Berlin, Germany
6 Center for Translational Medicine, Immunology, and Transplantation, Medical Department I, Marien Hospital Herne, University Hospital of the Ruhr-University Bochum, Hölkeskampring 40, 44625 Herne, Germany

* Corresponding Author, email address: manfred.gossen@hereon.de

[1] Corresponding author: Dr. Manfred Gossen, Institute of Active Polymers and Berlin-Brandenburg Center for Regenerative Therapies, Helmholtz-Zentrum Hereon, Kantstr. 55, 14513 Teltow, Germany. Tel.: +49 (0) 30 450539-491; Fax: +49 (0)30 450539-991; E-mail: manfred.gossen@hereon.de.

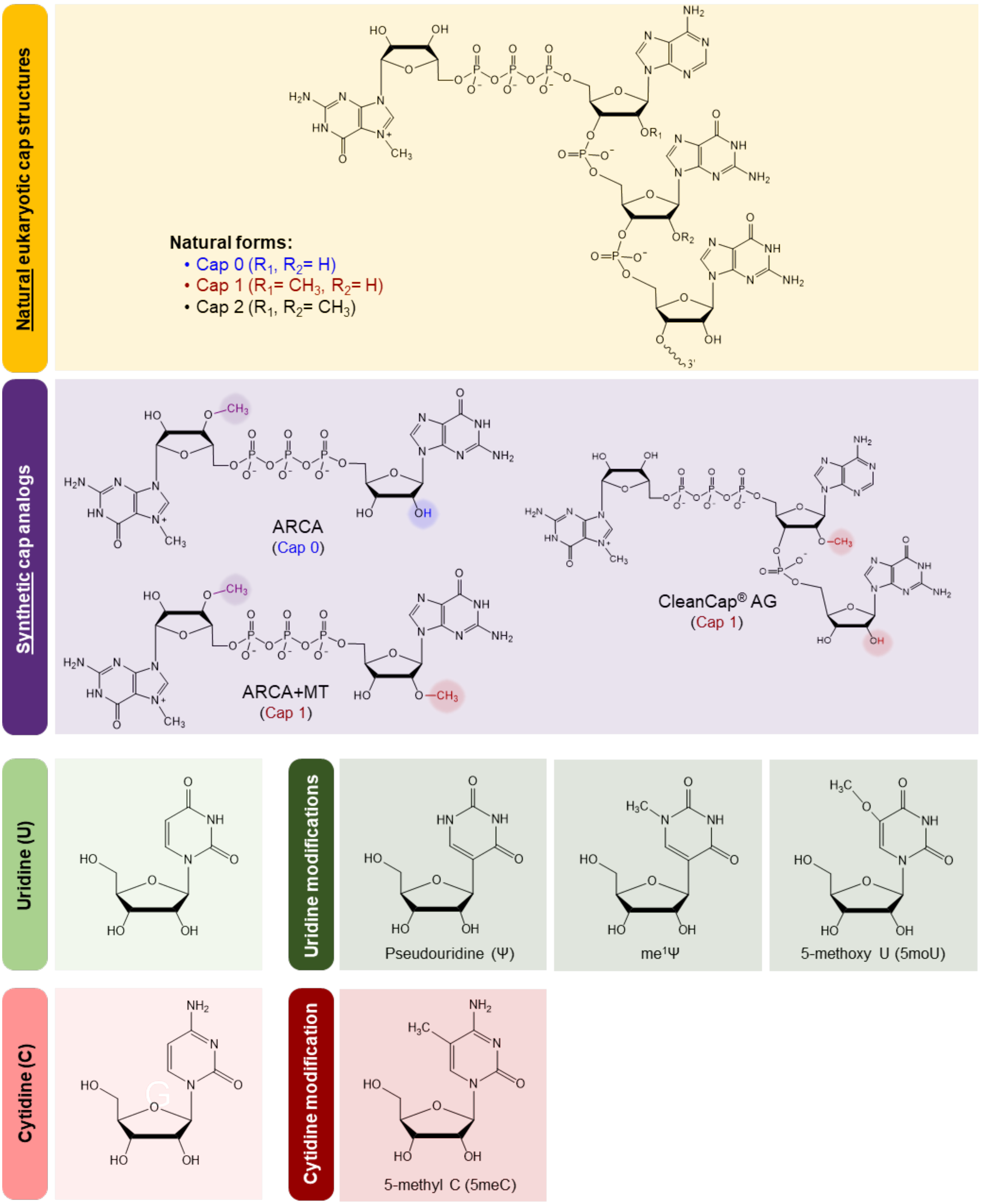

Figure S1. The upper panel (yellow box) depicts the chemical formula of naturally occurring eukaryotic cap structures in context of mRNA modification including Cap 0, Cap 1 and Cap 2 forms. Synthetic anti-reverse cap analogs used as initiator of *in vitro* mRNA synthesis process in form of dinucleotide ARCA, with and without extra methylation (Cap 0 vs. Cap 1), and trinucleotide CleanCap®AG, methylated at first adenosine (Cap1) (purple box). The changes of cap analogs compared to the natural cap are highlighted. The lower panels depict original and modified uridine and cytidine nucleoside implemented in this study; (ARCA: anti-reverse cap analogue, MT: methyl-transferase, Ψ: pseudouridine, me^{1}Ψ: N^1-Methylpseudouridine, 5moU: 5-methoxy-uridine, 5meC: 5-methyl-cytidine)

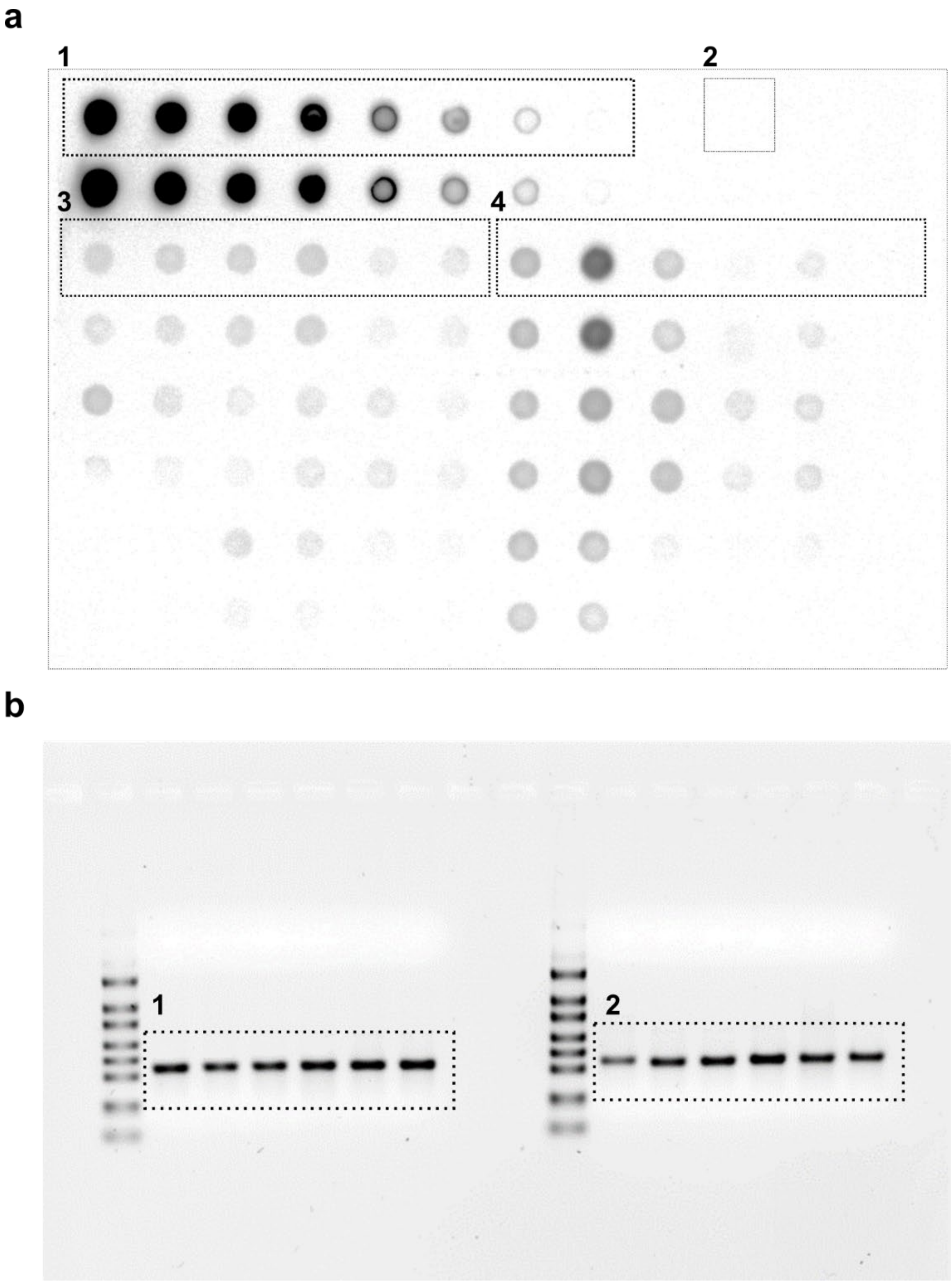

Figure S2. The uncropped image of **(a)** a dot blot membrane and **(b)** an agarose gel. **(a)** The dotted frames on dot blot correspond to samples as follows; 1) dsRNA standard series, 2) ssRNA as negative control, 3) cap modified IVT-mRNAs, 4) nucleotide modified IVT-mRNAs; all of which are presented in figure 3a-c. **(b)** The dotted frames on the agarose gel image correspond to 1) cap modified and 2) nucleotide modified IVT-mRNAs presented in figure 3b, c.

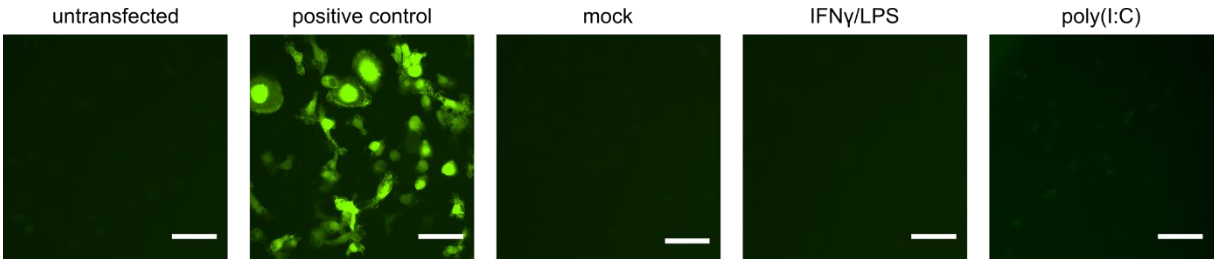

Figure S3. Fluorescent images of control samples, including untransfected, EGFP transfected positive control, mock transfected (LipoMM only), IFNγ/LPS treated as proinflammatory immune response positive control and poly(I:C) transfected macrophages as dsRNA-induced antiviral response positive control. Scale bar= 50 µm.

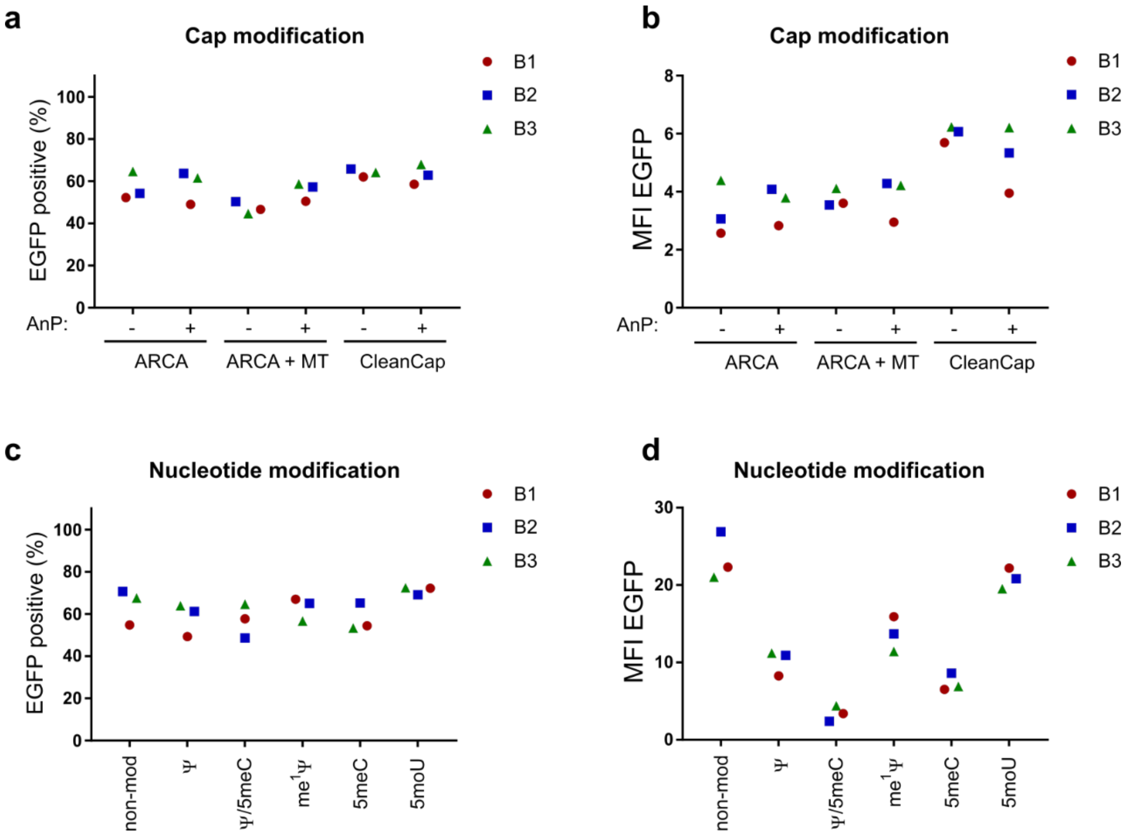

Figure S4. Evaluation of batch effects by comparison of three independently synthesized IVT-mRNA batches within the same donor; comparison of **(a)** transfection efficiency and **(b)** level of EGFP production between three batches of IVT-mRNA (B1-B3), with cap modification. Macrophages transfected IVT-mRNA with distinct nucleotide modification were evaluated in terms of **(c)** transfection efficiency and **(d)** level of EGFP production throughout three IVT-mRNA batches. (ARCA: anti-reverse cap analogue, MT:methyl-transferase, AnP: Antarctic phosphatase, Ψ: pseudouridine, me^{1}Ψ: N^1-Methylpseudouridine, 5moU: 5-methoxy-uridine, 5meC: 5-methyl-cytidine)

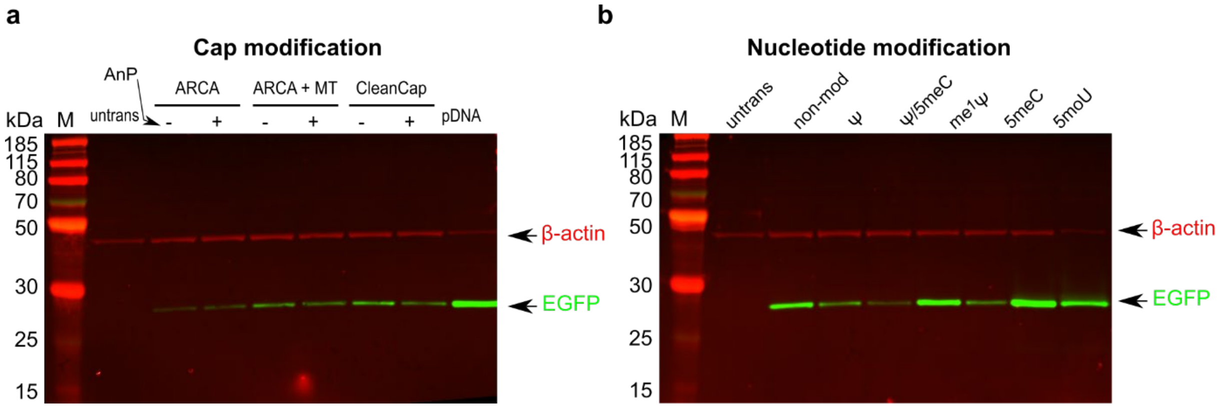

Figure S5. Protein expression evaluated by western blot analyses for macrophages transfected with either **(a)** cap modified or **(b)** nucleotide modified IVT-mRNA (ARCA: anti-reverse cap analogue, MT:methyl-transferase, AnP: Antarctic phosphatase, Ψ: pseudouridine, me^{1}Ψ: N^1-Methylpseudouridine, 5moU: 5-methoxy-uridine, 5meC: 5-methyl-cytidine)

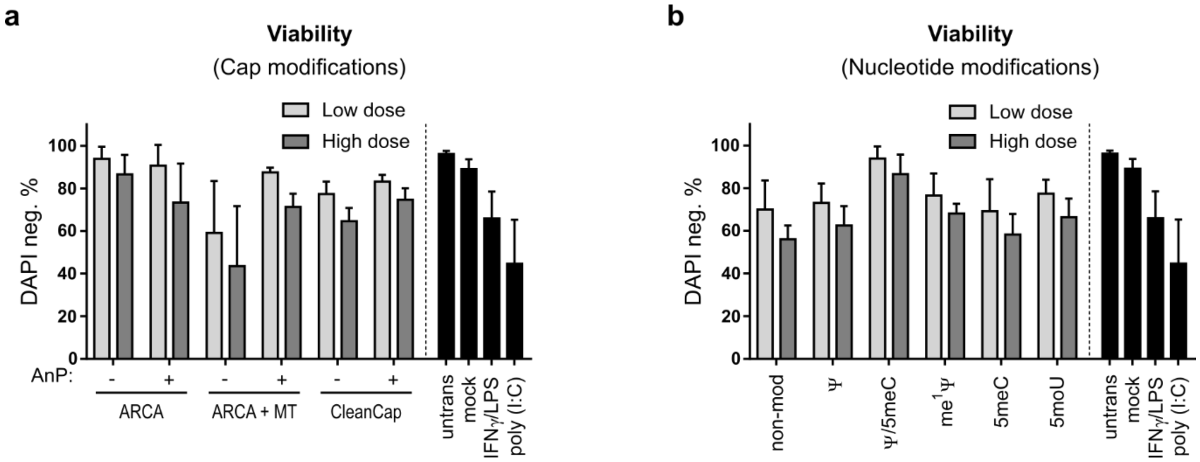

Figure S6. Viability of macrophages transfected with low dose and high dose of IVT-mRNA with chemical modification of either **(a)** cap structure or **(b)** nucleotides, evaluated by determining the percentage of DAPI-negative cells. Values are presented as mean ± SD, n = 3. (ARCA: anti-reverse cap analogue, MT: methyl-transferase, AnP: Antarctic phosphatase, Ψ: pseudouridine, me^{1}Ψ: N^1-Methylpseudouridine, 5moU: 5-methoxy-uridine, 5meC: 5-methyl-cytidine)

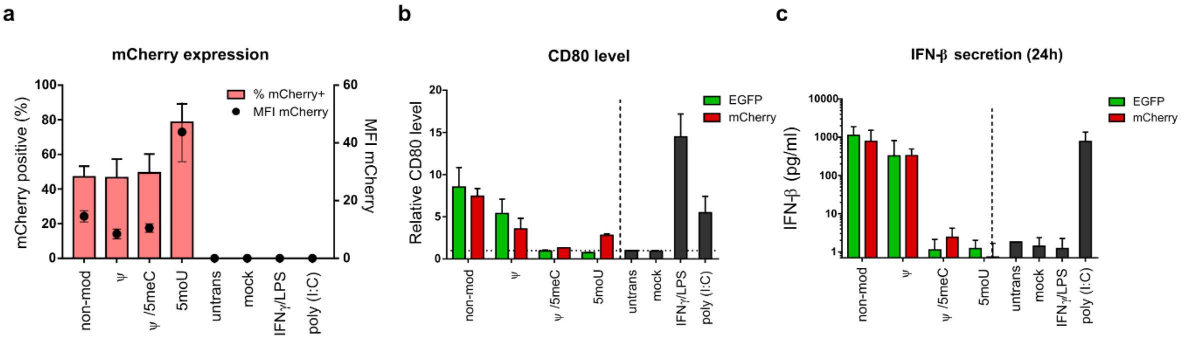

Figure S7. Macrophage response to transfection of mRNA coding for mCherry and EGFP with different nucleotide modification. (a) Transfection efficiency (bar graph, left axis), and mRNA expression level quantified by percentage of mCherry positive cells and mean fluorescent intensity of mCherry, respectively, (scattered dots, right axis), quantified using flow cytometry, 24 h post transfection. Comparison of CD80 level (b) and IFN-β secretion (c) by macrophages after IVT-mRNA transfection enconding for mCherry or EGFP with different nucleotide modifications, both measured 24 h after transfection. mCherry IVT-mRNA was transfected in low dose, i.e. 125 ng·mL^{-1}. Untransfected, poly(I:C)-treated, and LPS/IFN-γ-activated macrophages served as negative, positive and high control, respectively. Values are presented as mean ± SD. Error bars indicate SD of three independent experiments from three individual donors.

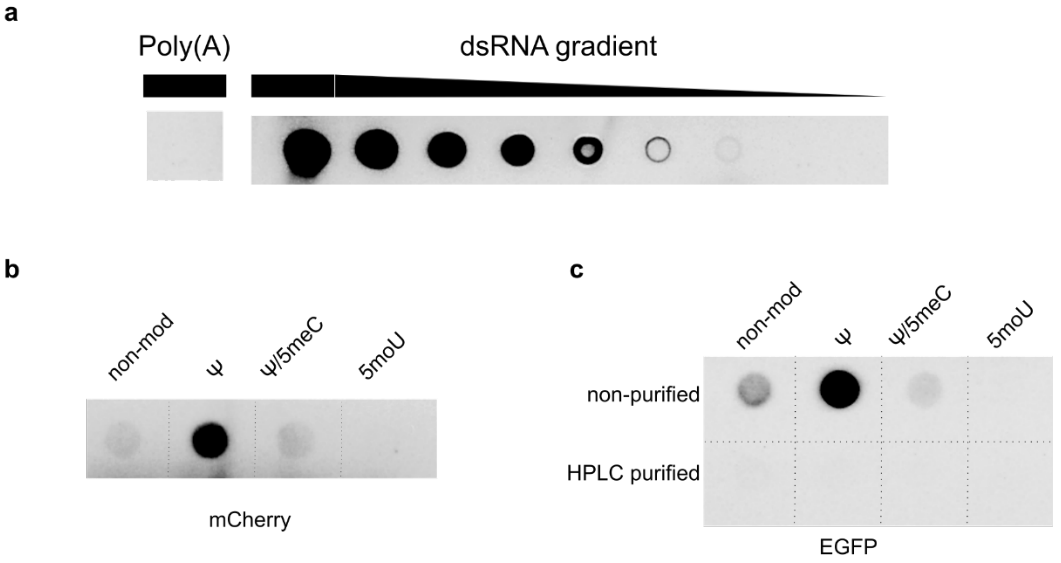

Figure S8. dsRNA detection by J2 antibody comparing non-purified with HPLC-purified IVT-mRNAs **(a)** Poly(A) as ssRNA negative control, and dsRNA positive control were blotted with the same amount as main samples (1000 ng/dot), dsRNA was 4-fold serial diluted (from left to right). **(b)** dsRNA content of mCherry encoding IVT-mRNA modified with different nucleotide modifications evaluated by J2 antibody binding. **(c)** Representative dot blots of EGFP encoding IVT-mRNAs with different nucleotide modifications comparing non-purified (top) versus HPLC-purified (bottom) samples. (Ψ: pseudouridine, 5moU: 5-methoxy-uridine, 5meC: 5-methyl-cytidine)

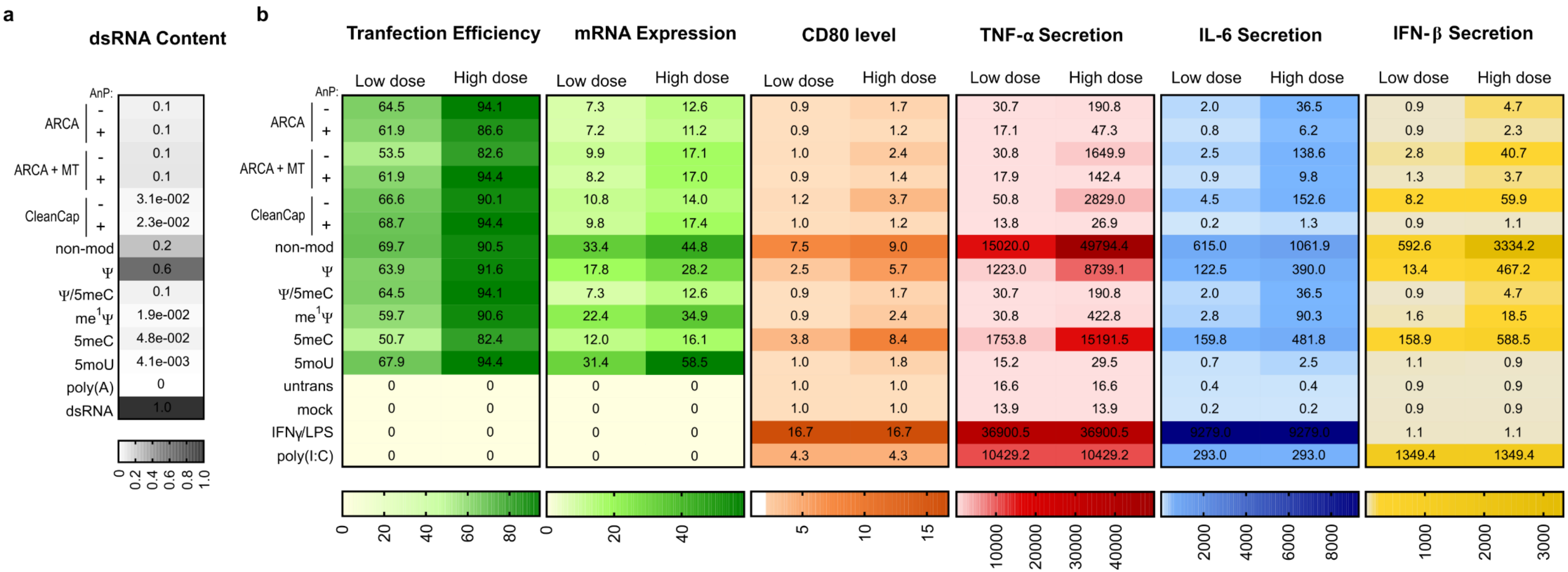

Figure S9. Overview of the properties and performance of IVT-mRNA with different cap and nucleotide modifications in terms of dsRNA content, protein expression, and inflammatory responses of transfected macrophages illustrated as heat map graphs. **(a)** dsRNA content, **(b)** (from left to right) transfection efficiency in percentage of EGFP positive cells, mRNA expression level measured by mean fluorescent intensity (MFI) of EGFP signal, and relative CD80 surface marker expression indicating the inflammatory responses, normalized MFI measured by flow cytometry. The cytokine secretion measured 24 h after transfection is presented as concentration (pg·mL^{-1}) of TNF-α, IL-6 and IFN-β. Untransfected, poly(I:C)-treated, and LPS/IFN γ-activated macrophages served as negative, positive and high control, respectively.

Table S1. Chemical modifications and process of preparation of IVT-mRNAs synthesized

	Sample ID	Chemical composition		Post-transcriptional modifications	
		Cap structure (type)	Nucleotides	2'-O-Methyltransferase treatment	Phosphatase treatment
**Cap modified IVT-mRNA**	ARCA/-	ARCA (Cap 0)	Ψ/5meC/A/G	-	-
	ARCA/+	ARCA (Cap 0)	Ψ/5meC/A/G	-	+
	ARCA+MT/-	ARCA (Cap 1)	Ψ/5meC/A/G	+	-
	ARCA+MT/+	ARCA (Cap 1)	Ψ/5meC/A/G	+	+
	CleanCap/-	CleanCap AG (Cap 1)	Ψ/5meC/A/G	-	-
	CleanCap/+	CleanCap AG (Cap 1)	Ψ/5meC/A/G	-	+
**Nucleotide modified IVT-mRNA**	non-mod	ARCA (Cap 0)	U/C/A/G	-	-
	Ψ	ARCA (Cap 0)	Ψ/C/A/G	-	-
	$me^1Ψ$	ARCA (Cap 0)	$me^1Ψ$/C/A/G	-	-
	5meC	ARCA (Cap 0)	U/5meC/A/G	-	-
	5moU	ARCA (Cap 0)	5moU/C/A/G	-	-
	Ψ/5meC (2nt. mod.)	ARCA (Cap 0)	Ψ/5meC/A/G	-	-

Sample ID refers to abbreviations which was used in text throughout. (ARCA: anti-reverse cap analogue, MT: methyl-transferase, Ψ: pseudouridine, $me^1Ψ$: N^1-Methylpseudouridine, 5moU: 5-methoxy-uridine, 5meC: 5-methyl-cytidine, 2nt. mod: two nucleotides were modified)
