# Supplementary material for: Chemical modification of uridine modulates mRNA-mediated proinflammatory and antiviral response in primary human macrophages
Source: Mol Ther Nucleic Acids. 2022 Jan 10;27:854–69. doi: 10.1016/j.omtn.2022.01.004 (PMC8807976; doi:10.1016/j.omtn.2022.01.004)
Supplement: Document S2. Article plus supplemental information [file mmc2.pdf]

Molecular Therapy
Nucleic Acids
Original Article

Chemical modification of uridine modulates mRNA-mediated proinflammatory and antiviral response in primary human macrophages

Hanieh Moradian,[1,2,3] Toralf Roch,[4,5,6] Larissa Anthofer,[1,2] Andreas Lendlein,[1,2,3] and Manfred Gossen[1,2]

[1]Institute of Active Polymers, Helmholtz-Zentrum Hereon, Kantstr. 55, 14513 Teltow, Germany; [2]Berlin-Brandenburg Center for Regenerative Therapies (BCRT), Föhrerstr. 15, 13353 Berlin, Germany; [3]Institute of Biochemistry and Biology, University of Potsdam, Karl-Liebknecht-Str. 24-25, 14476 Potsdam, Germany; [4]Berlin Institute of Health at Charité – Universitätsmedizin Berlin, BIH Center for Regenerative Therapies (BCRT), Charitéplatz 1, 10117 Berlin, Germany; [5]Charité – Universitätsmedizin Berlin, Corporate Member of Freie Universität Berlin, Humboldt-Universität zu Berlin, Berlin Center for Advanced Therapies, Augustenburger Platz 1, 13353 Berlin, Germany; [6]Center for Translational Medicine, Immunology, and Transplantation, Medical Department I, Marien Hospital Herne, University Hospital of the Ruhr-University Bochum, Hölkeskampring 40, 44625 Herne, Germany

***In vitro* transcribed (IVT)-mRNA has been accepted as a promising therapeutic modality. Advances in facile and rapid production technologies make IVT-mRNA an appealing alternative to protein- or virus-based medicines. Robust expression levels, lack of genotoxicity, and their manageable immunogenicity benefit its clinical applicability. We postulated that innate immune responses of therapeutically relevant human cells can be tailored or abrogated by combinations of 5′-end and internal IVT-mRNA modifications. Using primary human macrophages as targets, our data show the particular importance of uridine modifications for IVT-mRNA performance. Among five nucleotide modification schemes tested, 5-methoxy-uridine outperformed other modifications up to 4-fold increased transgene expression, triggering moderate proinflammatory and non-detectable antiviral responses. Macrophage responses against IVT-mRNAs exhibiting high immunogenicity (e.g., pseudouridine) could be minimized upon HPLC purification. Conversely, 5′-end modifications had only modest effects on mRNA expression and immune responses. Our results revealed how the uptake of chemically modified IVT-mRNA impacts human macrophages, responding with distinct patterns of innate immune responses concomitant with increased transient transgene expression. We anticipate our findings are instrumental to predictively address specific cell responses required for a wide range of therapeutic applications from eliciting controlled immunogenicity in mRNA vaccines to, e.g., completely abrogating cell activation in protein replacement therapies.**

INTRODUCTION

Growing demands for rapid, robust, and scalable production of therapeutics for disease prevention or treatment lead to remarkable advances in mRNA-based medicines over the past few years.[1–3] Lack of genotoxicity and facile production, as well as efficient intracellular delivery are advantages of mRNA therapeutics, when compared with preceding non-cellular, nucleic acids-based Advanced Therapy Medicinal Products such as recombinant viruses of DNA or recombinant protein-based medicines.[4,5] Clinical applications of mRNA include both, protein replacement therapies[6] and mRNA vaccines,[7,8] deployed not only for treatment of inherited and non-infectious acquired diseases such as cancer,[9] but also viral diseases, such as recently the severe acute respiratory syndrome coronavirus 2 (SARS-CoV-2).[10,11] The latter is a showcase example for the power of mRNA technology in tackling disease, outpacing other types of vaccines, with rather fast development from bench to market.[12]

Despite progress in mRNA production technology by *in vitro* transcription (IVT) via bacteriophage enzymes such as SP6, T3, and T7 RNA polymerases, potential immunogenicity of transcripts remains a major issue for some mRNA-based medicines.[5,13] The exogenous *in vitro* transcribed mRNAs (IVT-mRNA) can be recognized by various endosomal and cytosolic pattern recognition receptors (PRRs).[8] Examples are Toll-like receptor-7, and -8 (TLR-7, -8) and TLR-3,[14] sensing single- and double-stranded RNA (ssRNA and dsRNA), respectively. The latter can also be recognized by melanoma differentiation-associated protein 5 (MDA5)[15,16] and retinoic acid-inducible gene I (RIG-I), which are part of the RIG-I-like receptor family. Pathways activated by these PRRs induce production of cytokines, such as type I interferons (IFNs), tumor necrosis factor-α (TNF-α), interleukin (IL)-1β, and IL-6,[17] as well as upregulation of co-stimulatory molecules such as CD80, CD86, and CD40.[18] RIG-I also recognizes the 5′-triphosphate end of IVT-RNA, particularly those of dsRNA termini.[19] In addition, cell-autonomous mechanisms mediated by 2′–5′ oligoadenylate synthases (OAS), RNase L, or the

Received 4 June 2021; accepted 7 January 2022;
https://doi.org/10.1016/j.omtn.2022.01.004.

Correspondence: Dr. Manfred Gossen, Institute of Active Polymers and Berlin-Brandenburg Center for Regenerative Therapies, Helmholtz-Zentrum Hereon, Kantstr. 55, 14513 Teltow, Germany.
E-mail: manfred.gossen@hereon.de

 © 2022 Helmholtz-Zentrum Hereon.
This is an open access article under the CC BY license (http://creativecommons.org/licenses/by/4.0/).

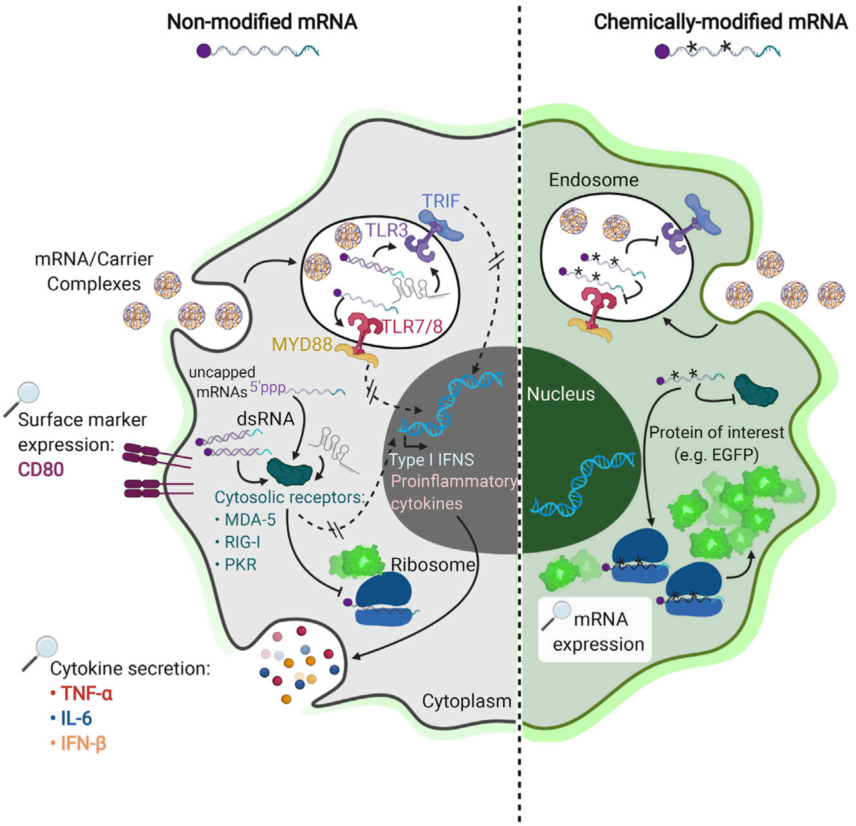

Figure 1. The effect of chemical modification of IVT-mRNA on potential cell response

Intracellular pathways of innate immune response in macrophages transfected with either non-modified (left) versus chemically modified IVT-mRNA (right). The elements labeled with magnifying glass are the actual readouts measured at the present study.

IFN-induced, RNA-activated protein kinase R (PKR) can directly lead to RNA instability and inhibition of RNA expression.[20–22]

Previous studies suggested several approaches to abrogate or modulate unintended immune activation, such as chemical modification of either cap structures[23,24] or nucleotides,[23–27] optimization of pDNA template sequence,[23,28] and modification of IVT reaction conditions,[29,30] as well as extra purification steps to remove impurities, e.g., dsRNA by-products.[31,32] However, most of these strategies were evaluated either *in vitro*, using non-primary macrophage and monocyte cell lines such as RAW 264.7 or THP-1, respectively, or investigated *in vivo* by using mouse models.[33] Indeed, macrophages are of particular interest for this type of study. Here, we analyze the effects of IVT-mRNA transfection in primary human monocyte-derived macrophages, which are the first line of cellular defense due to their high phagocytosis capacity. We, therefore, consider this cell type to be of special relevance for clinical research, not only for its expected uptake of formulated IVT-mRNA even if not specifically targeted,[34] but also because of its considerable immune-modulatory capacity,[35,36] as well as its ability to initiate and modulate antiviral or anti-tumor T cell responses[37,38] as antigen-presenting cells,[39] and as a potential direct target in addressing macrophage-related diseases.[40]

We postulated that systematic analysis of different 5′-end and internal nucleotide modifications of IVT-mRNA transfected in human macrophages reveals the pattern of cellular response, which could be harnessed to minimize or potentially abrogate the subsequent immune response (Figure 1). In a comprehensive side-by-side study, mRNA constructs with three distinct cap structures, with or without extra phosphatase treatment, were investigated in parallel with three different uridine modifications and one cytidine modification; see Figure 2 and Table S1 for details of mRNA synthesis process and chemistry, and Figure S1 for precise chemical formula of cap and nucleoside modifications. The quality and biological performance of chemically modified IVT-mRNAs upon transfection in macrophages were evaluated as key readouts. This was achieved by assessing changes in surface marker expression and the cytokine secretion patterns as indicators of antiviral and proinflammatory immune responses. Transfection efficiency and level of transgene expression were determined to rule out that the postulated effect of modifications, i.e., dampening of the innate immune response, should not reduce protein production. In fact, the highest possible level of expression from the administration of the lowest feasible dose of mRNA is desired, if not required, for most clinical applications, also under health economic considerations. Thus, our work provides guidelines to uncouple maximized IVT-mRNA-mediated protein production levels from immune activation that could be prohibitive for future translational applications.

RESULTS

Study design

IVT-mRNAs with distinct chemical compositions were evaluated by agarose gel electrophoresis and dot blot analysis to determine transcript integrity and potential dsRNA by-products, respectively. Primary human macrophages were generated from blood-derived $CD14^+$ monocytes. Macrophages (MΦs) were subsequently transfected with lipoplexes containing the chemically modified IVT-mRNAs. The fluorescent protein marker production was evaluated 24 h posttransfection by fluorescent microscopy and further quantified with flow cytometry. In parallel, immune response of transfected MΦs was assessed either by measurement of activation-induced cell surface molecules such as CD80, and by analyzing cytokine secretion patterns, which included TNF-α, IL-6, and IFN-β.

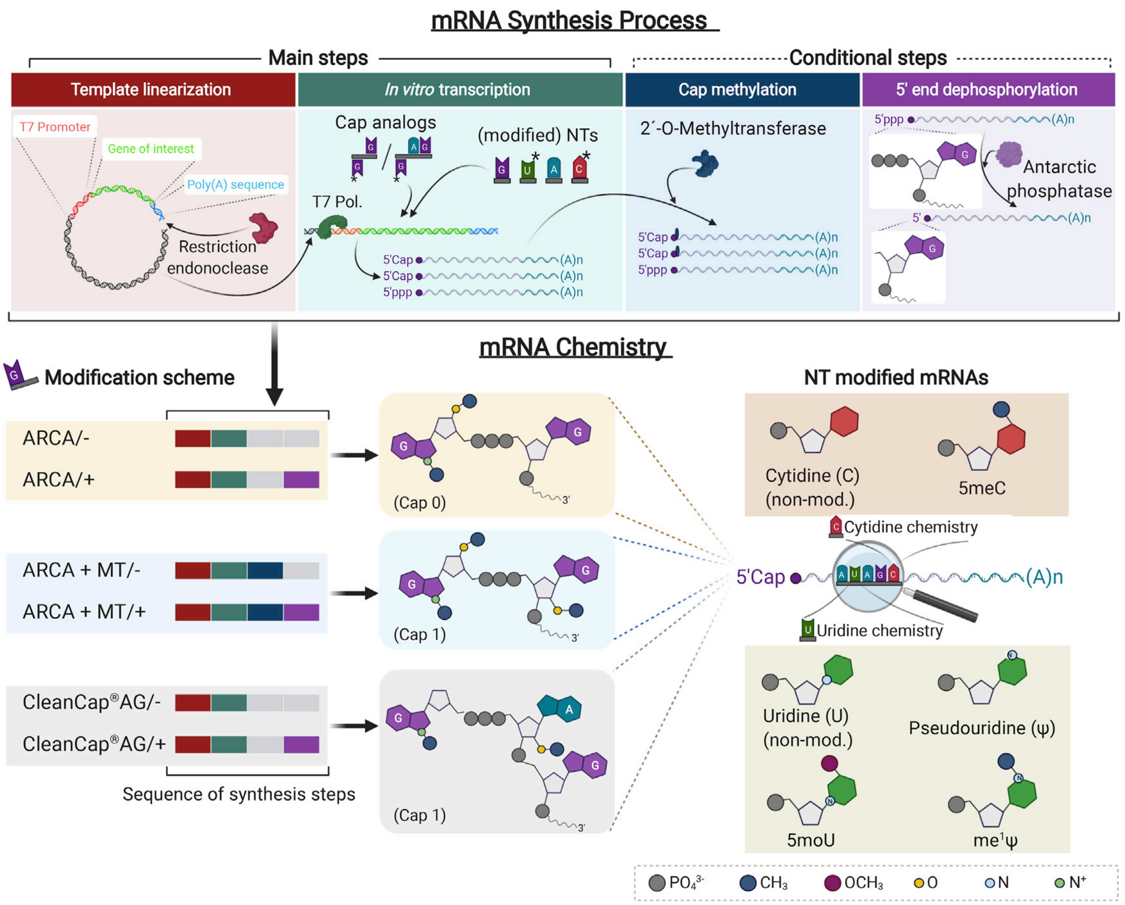

Figure 2. Overview of *in vitro* transcription process and chemical modifications of IVT-mRNA used in this study

Main steps, and conditional post-transcriptional treatments, which were only applied to samples with cap modifications, i.e., methylation and dephosphorylation, are illustrated at the top panel. Simplified chemistry of IVT-mRNA synthesized and utilized at this study with various cap and/or nucleotide modifications are depicted at the bottom panel. Sequence of synthesis steps applied for cap modified mRNAs are indicated as colored bars corresponding to each synthesis step. Phosphatase treatment step is further indicated with/– or/+ next to sample name. Chemical formula only indicate the main variations among nucleotides as well as cap structures. See Figure S1 for precise chemical formula of cap and nucleotide modifications, and Table S1 for detailed information on chemistry and synthesis process of each sample. pDNA, plasmid DNA; T7 Pol., T7 RNA polymerase; NTs, nucleotides; ARCA, anti-reverse cap analog; Ψ, pseudouridine; me^{1}Ψ, N^1-Methylpseudouridine; 5moU, 5-methoxy-uridine; 5meC, 5-methyl-cytidine.

Chemical modification of IVT-mRNA

Three variations of cap structure, including anti-reverse cap analogs (ARCA) as an example of Cap 0 structure, 2′-O-methylated ARCA (ARCA + MT), and CleanCap AG (CleanCap), as two examples of Cap 1 structure, were investigated side-by-side (Figure 2). Note that co-transcriptional integration of CleanCap requires a nucleotide change in T7 promoter at +1/+2 positions from "GG" to "AG"; see "Materials and methods: IVT-mRNA synthesis with various chemical modifications" for detailed explanation of the procedure.

Since none of the co-transcriptional cap modifications are entirely efficient in transcript capping, a fraction of uncapped IVT-mRNA could trigger immune response through their 5′-triphosphate end groups. Thus, to investigate the effect/necessity of 5′-triphosphate removal on overall biological performance of IVT-mRNA products, both in terms of protein production level and immune stimulation, an extra phosphatase treatment was included for each of the three examined cap structures and evaluated in parallel.

To assess the importance of IVT-mRNA nucleotide modifications, non-modified IVT-mRNA was compared with uridine modifications, namely pseudouridine (Ψ), N1-methyl-pseudouridine (me^{1}Ψ), and 5-methoxyuridine (5moU), a cytidine modification 5-methylcytidine (5meC), as well as a combination of Ψ and 5meC (Ψ/5meC) (Table S1).

Of note, IVT-mRNA synthesized with the various cap structures for comparative analysis of the effect of 5′-end modifications were uniformly substituted with Ψ and 5meC. This combination of nucleotide modifications, which has been extensively analyzed in the past, is

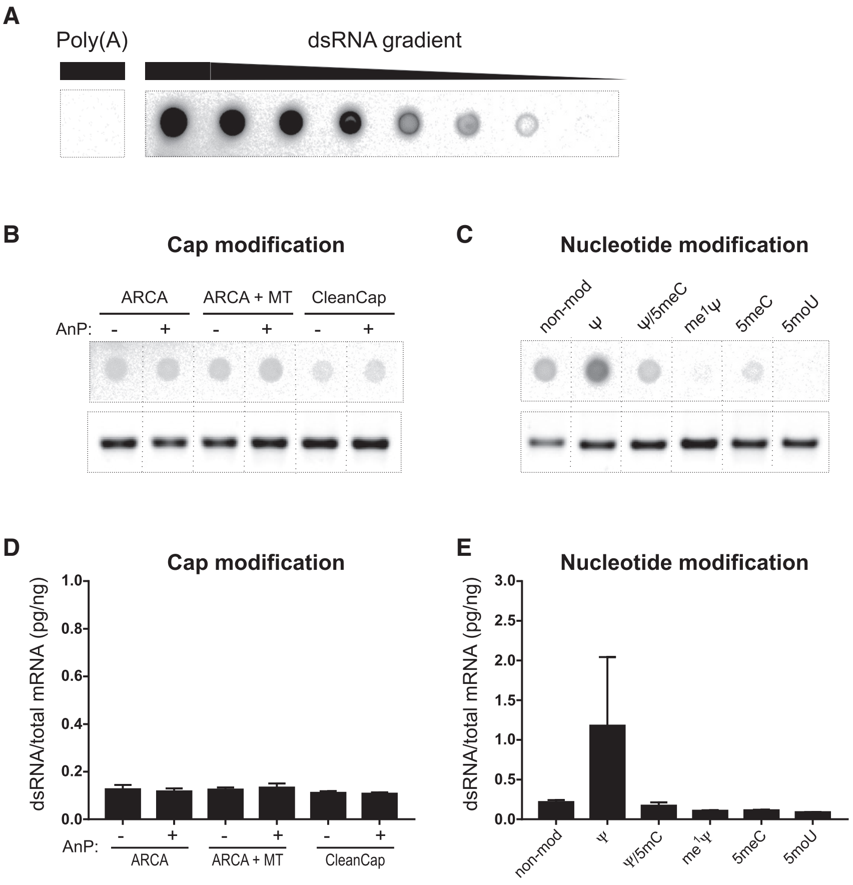

Figure 3. IVT-mRNA double strand content and integrity investigated by dot blot and agarose gel electrophoresis

(A) Poly(A) as ssRNA-negative control, and dsRNA positive control were blotted with the same amount as main samples (1,000 ng/dot), next to dsRNA gradient of 4-fold serial dilutions for generating a standard curve for subsequent quantifications and detected by a dsRNA-antibody. Representative dot blots of IVT-mRNAs with different (B) cap modifications, and (C) nucleotide modifications presented side-by-side with denatured agarose gel electrophoresis images of the same samples. Quantified dsRNA for IVT-mRNAs with various cap modifications (D) as well as (E) nucleotide modifications plotted as weight percent of dsRNA content (calculated according to positive control standard curve) to total mRNA amount blotted on membrane for each sample. Error bars indicate SEM for three independently synthesized IVT-mRNA batches blotted on the membrane in duplicates; see Figure S2 for uncropped membrane and the gel image. ARCA, anti-reverse cap analog; MT, methyl-transferase; AnP, Antarctic phosphatase; Ψ, pseudouridine; me^{1}Ψ, N^1-Methylpseudouridine; 5moU, 5-methoxy-uridine; 5meC, 5-methylcytidine.

known to reduce the immune response without its complete elimination,[41] and thus can serve as a baseline for analyzing further modifications. Conversely, for comparatively analyzing the effects of internal nucleotide modifications, we uniformly incorporated a 5′ ARCA as a standard cap structure in the synthesis of IVT-mRNAs.

Chemical modification of nucleotides, but not cap structure, affects dsRNA content of IVT-mRNA

Dot blot analysis was performed using the J2 dsRNA-specific antibody in order to evaluate the degree of dsRNA formation in the run-off transcripts, which is a major trigger of cellular anti-IVT-mRNA responses (Figure 3), as positive control serial dilutions of a dsRNA sample were used for validation and subsequent quantification (Figure 3A). Accordingly, the calculated values of dsRNA were normalized to the total amount of membrane-immobilized IVT-mRNA for each sample. Identical amounts of ssRNA were measured as negative control, and found to be non-detectable by the dsRNA-specific antibody, ruling out the interference of unspecific binding of J2 antibody in this experimental setup (Figure 3A).

No obvious differences were observed between signal intensities of IVT-mRNAs equipped with different cap structures, in the groups with or without 5′-end dephosphorylation (Figure 3B). This finding was also proved quantitatively, with only minor variations in dsRNA content (Figure 3D). In contrast, IVT-mRNAs with ARCA as cap structure and various nucleotide modifications had a prominent effect on dsRNA content of IVT-mRNA products. Notably, the highest dsRNA content was found in non-modified IVT-mRNA, and Ψ-modified IVT-mRNA. However, the dsRNA signal was reduced by 5meC, and Ψ/5meC modifications of IVT-mRNAs. Interestingly, IVT-mRNA with other uridine modifications, i.e., me^{1}Ψ and 5moU, resulted in the lowest number of dsRNA by-products (Figures 3C and 3E), underscoring overall impact of uridine on quality of transcripts. Moreover, the integrity of IVT-mRNA samples was evaluated with agarose gel electrophoresis (Figures 3B and 3C) to rule out correlation of detected dsRNA signal to presence of potential unknown side-products or possible degradation. Of note, similar patterns were consistently observed throughout IVT-mRNA samples of three independent syntheses.

Protein production level was substantially influenced by IVT-mRNA chemistry

Transfection efficiency and level of protein production were measured as key parameters to assess biological performance of IVT-mRNAs with distinct chemical modifications. IVT-mRNA coding enhanced GFP (EGFP) was transfected in primary human monocyte-derived macrophages in low dose (125 ng·mL^{-1}) and high dose (500 ng·mL^{-1}). The corresponding mRNA amounts for the two administrated doses were selected according to cell viability and immune activation as described in our earlier study.[41] IVT-mRNA expression was initially assessed qualitatively by fluorescent microscopy (Figures 4A and 4E), and quantified at single cell resolution by

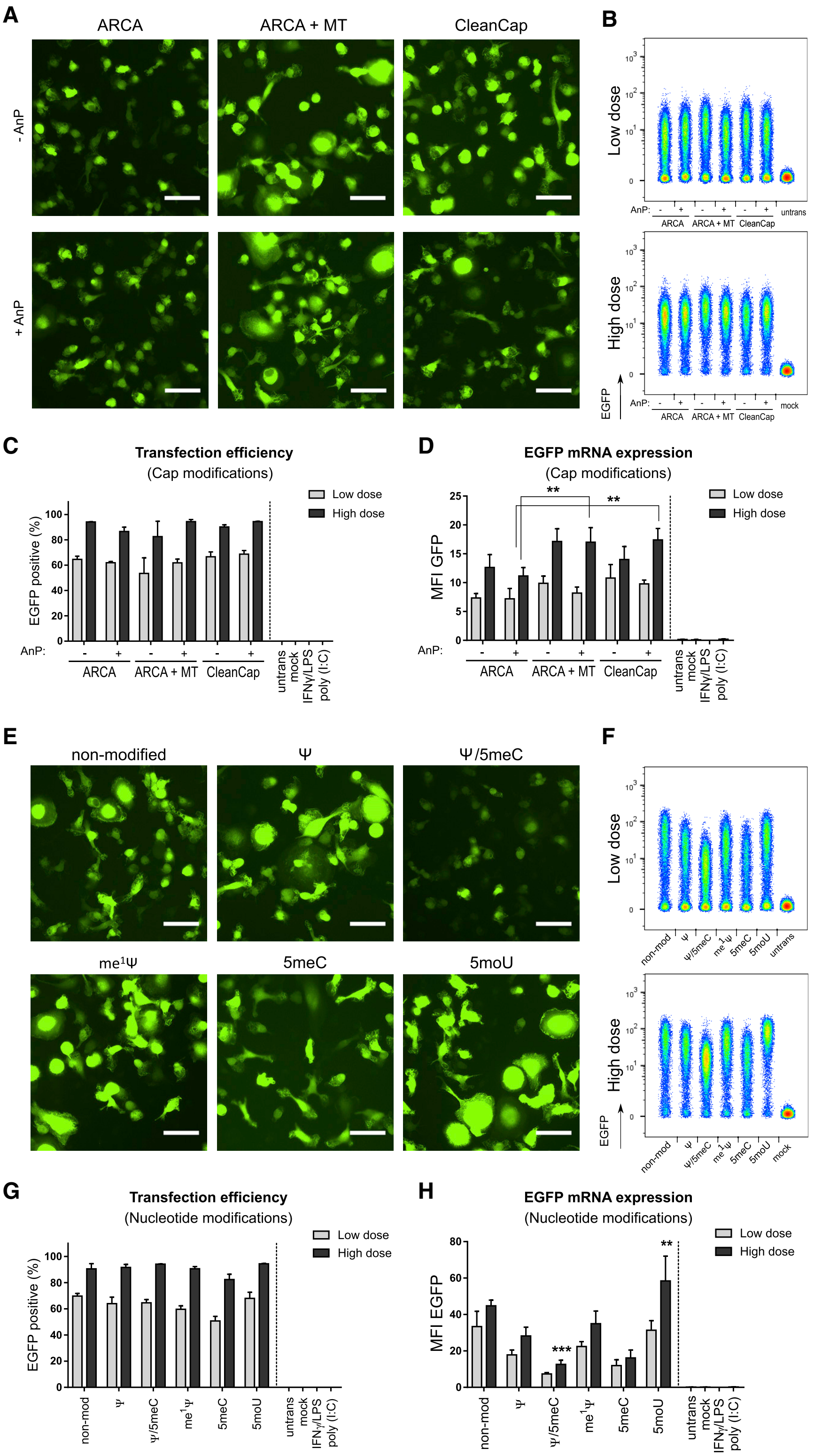

Figure 4. Transfection efficiency and EGFP mRNA expression level in macrophages transfected with IVT-mRNA with various cap and nucleotide modifications

(A) Representative fluorescent images and (B) flow cytometric density plots indicating EGFP expression in macrophages transfected with low dose and high dose of IVT-mRNA made of either Cap 0 (i.e., ARCA), or Cap 1 (i.e., ARCA + MT, and CleanCap) with and without phosphatase treatment. (C) Quantification of transfection efficiency, and (D) EGFP mRNA expression level in macrophages transfected with low doses and high doses of the different IVT-mRNAs with cap modifications. (E) Representative fluorescent images of macrophages transfected with low doses of IVT-mRNA composed of nucleotides with different chemical modifications. (F) Flow cytometric density plots indicating EGFP expression in macrophages transfected with low dose and high dose of IVT-mRNA with various nucleotide modifications. (G) Transfection efficiency and (H) EGFP mRNA expression level quantified by flow cytometry and plotted in terms of EGFP-positive cells percentage and MFI of EGFP signal among EGFP-positive cell populations, respectively. Poly(I:C) was also transfected in low dose (125 ng$\cdot$mL^{-1}). For each condition 125 ng$\cdot$mL^{-1} and 500 ng$\cdot$mL^{-1} of IVT-mRNA were used for transfection referred here as low dose and high dose, respectively. Values are presented as mean ± SD. Error bars indicate SD of three independent experiments from three individual donors. Bar = 50 μm. Statistical differences are depicted with $^{**}p < 0.005$, $^{***}p < 0.001$. ARCA, anti-reverse cap analog; MT, methyl-transferase; AnP, Antarctic phosphatase; Ψ, pseudouridine; me^{1}Ψ, N^1-Methylpseudouridine; 5moU, 5-methoxy-uridine; 5meC, 5-methyl-cytidine.

flow cytometry (Figures 4B–4D, 4F–4H). Modifications of the cap structure had only a slight impact on fluorescence intensity of macrophages transfected with either Cap 0 (ARCA) or Cap 1 (ARCA + MT or CleanCap) modified IVT-mRNAs (Figures 4A and 4B). In addition, no obvious differences in EGFP production from phosphatase-treated IVT-mRNAs, for any of the three examined cap structures could be observed (Figure 4A). No signal was detected for the untransfected controls (Figure S3). Moreover, flow cytometric assessment revealed substantial amounts of EGFP-producing MΦ with slight variations in EGFP production level after transfection with low as well as high doses of cap modified IVT-mRNA (Figure 4B). Transfection efficiency and EGFP mRNA expression was quantified as percent of EGFP-positive cells, and mean fluorescent intensity (MFI) of EGFP in positive cells, respectively (Figures 4C and 4D). Remarkably, the low and high IVT-mRNA doses led to a transfection efficiency of more than 60% and 80%, respectively. However, no significant differences were found between transfection efficiency of IVT-mRNAs with distinct cap modifications, neither at low dose nor at high dose (Figure 4C). When treated with phosphatase, the level of EGFP production was significantly higher for Cap 1 (i.e., ARCA + MT and CleanCap) compared with Cap 0 (i.e., ARCA) at high dose of mRNA (Figure 4D).

For IVT-mRNA with chemical modifications of nucleotides, non-modified as well as Ψ, $me^1Ψ$, and 5moU modified IVT-mRNAs led to the highest EGFP signal intensity, notably also at low dose of mRNA, whereas 5meC and its combination with Ψ resulted in the lowest EGFP synthesis in transfected MΦs, measured 24 h post transfection (Figure 4E). Different EGFP levels were consistently detected in MΦs transfected with various nucleotide modifications both at low dose and, more prominently, at high dose of IVT-mRNA (Figure 4F). Especially 5moU outperformed the other chemical modifications under the aspect of maximizing protein synthesis. While the number of EGFP-positive cells was not affected by different nucleotide modifications at low and high doses of mRNA (Figure 4G), the EGFP production level was substantially higher for 5moU, non-modified, and $me^1Ψ$ IVT-mRNA, especially at high dose of mRNA (Figure 4H). The lowest levels of EGFP mRNA expression were consistently observed for Ψ/5meC modified IVT-mRNA (Figures 4E, 4F, and 4H). The results were consistent for different mRNA syntheses, since similar transfection efficiencies and EGFP intensities could be observed when one donor was treated with three independently prepared IVT-mRNA batches (Figure S4), which excluded a potential bias due to batch effects.

In addition to the quantitative assessment with flow cytometry and qualitative analysis with fluorescent microscopy, the resulting protein produced by IVT-RNA with different chemical modifications were evaluated using western blot analysis. In fact, it could be confirmed that intact EGFP protein was produced for all chemically modified IVT-mRNA with no sign of other unspecific side products (Figure S5).

Nucleotide chemical modifications of IVT-mRNA modulate CD80 in transfected MΦs already at low doses

Unintended cellular stress, and immune responses elicited by IVT-mRNA upon transfection are critical issues, which could lead to complete inhibition of protein production machinery and eventually result in cell death. Analysis of co-stimulatory surface molecules, such as CD80, was found to be a valuable readout for evaluating the activation of transfected MΦs.[41] Thus, CD80 production was measured by flow cytometry and compared within different IVT-mRNA modifications (Figure 5). Macrophage treatment with IFNγ/lipopolysaccharide (LPS) resulted in a substantial upregulation of CD80, whereas poly(I:C) induced only little amounts of CD80. Interestingly, the CD80 levels on cells transfected with low dose of IVT-mRNA remained unchanged, irrespective of cap modifications, whereas noticeable CD80 upregulations were detected at the high-dose conditions (Figure 5A). Quantification of the results revealed that phosphatase treatment consistently reduced these elevated CD80 levels for all three examined cap structures (Figure 5B).

Nucleotide modification of IVT-mRNA, on the other hand, resulted in pronounced differences in CD80 level both at low dose, and more dramatically at high dose of IVT-mRNA transfected MΦs, clearly recognizable by comparison of histograms with negative and positive controls (Figure 5C). Maximum level of CD80 was related to non-modified and 5meC-modified IVT-mRNA, when compared quantitatively (Figure 5D). While Ψ modification resulted in high CD80 production levels, combined Ψ and 5meC modifications resulted in substantial reduction of IVT-mRNA induced CD80 production levels (Figure 5D). Other uridine modifications including $me^1Ψ$ or 5moU led to no changes in CD80 production in relation to untransfected MΦs when transfected at low dose of IVT-mRNA, and only a slight increase at high dose (Figure 5D).

The downstream effect of IVT-mRNA-induced immune response on cell viability was investigated by measurement of DAPI-negative cells via flow cytometry. Interestingly, MΦs that were producing a higher level of CD80, such as high dose of ARCA + MT in cap modified IVT-mRNA and non-modified, Ψ, and 5meC-modified IVT-mRNAs, were observed to have a low level of cell viability (Figure S6).

Chemical modifications of IVT-mRNA influenced both proinflammatory and antiviral cytokines secretion by transfected MΦs

To evaluate the immune activation of MΦs, secretion of TNF-α, IL-6, and IFN-β was measured at 6 h and 24 h posttransfection, throughout all cap modifications (Figure 6), as well as nucleotide modifications (Figure 7). There were no significant differences between TNF-α and IL-6 secretion from MΦs transfected with Cap 0 structure (i.e., ARCA) and Cap 1 structures (i.e., ARCA + MT, or CleanCap) 6 h after transfection (Figures 6A and 6C). This applies for low dose and most of high doses of IVT-mRNA, in particular when absolute cytokine levels are considered in relation to the LPS positive control. However, after 24 h, Cap 1 structures induced higher levels of TNF-α and IL-6 compared with Cap 0 at high dose of mRNA (Figures 6B and 6D). Noteworthy, phosphatase-treated IVT-mRNAs elicited less TNF-α and IL-6 secretion at high dose of IVT-mRNA, consistently for all cap structures, when compared with untreated IVT-mRNA of the same cap formula

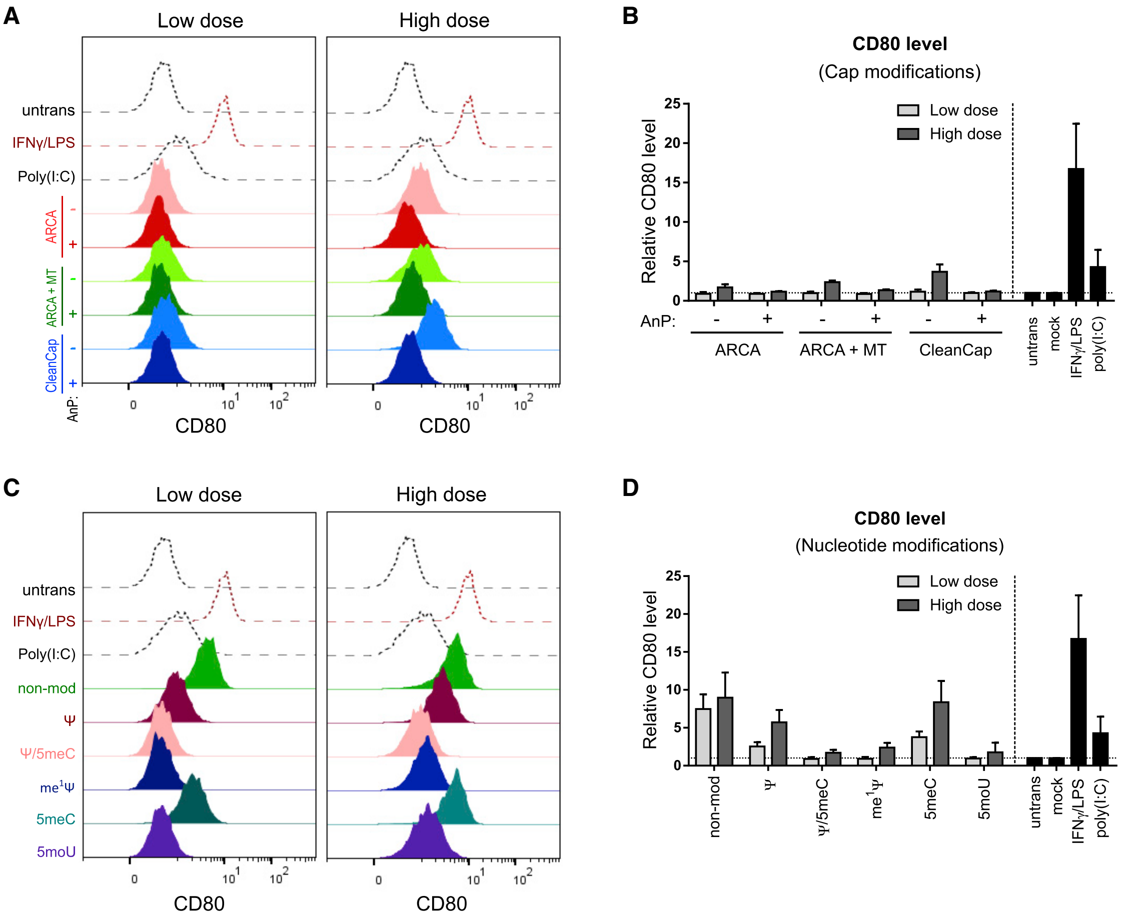

Figure 5. Evaluation of CD80 expression in macrophages, in response to IVT-mRNA transfection with different chemistry

Staggered histogram of CD80 levels in macrophages transfected with low doses and high doses of IVT-mRNA with various cap modifications (A), as well as nucleotide modifications (C) along with untransfected, poly(I:C), the dsRNA positive control transfected in low dose, and activated macrophages. Activated cells were treated with LPS/IFN-γ. MFI of CD80 normalized to untransfected cells is indicated for macrophages transfected with low doses and high doses of mRNA with different cap modifications (B) and nucleotide modifications (D). Mock transfection refers to carrier (i.e., LipoMM) without mRNA. For each condition 125 ng • mL^{-1} and 500 ng • mL^{-1} of IVT-mRNA were used for transfection, referred here as low dose and high dose, respectively. Expression was measured 24 h after transfection. Values are presented as mean ± SD. Error bars indicate SD of three independent experiments from three individual donors (n = 3). ARCA, anti-reverse cap analog; MT, methyl-transferase; AnP, Antarctic phosphatase; Ψ, pseudouridine; $me^1Ψ$, N^1-Methylpseudouridine; 5moU, 5-methoxy-uridine; 5meC, 5-methyl-cytidine.

(Figures 6A–6D). Similar patterns were observed for IFN-β, as enhanced IFN-β secretion was detected for Cap 1 compared with Cap 0 (Figures 6E and 6F). While there were no remarkable differences between various cap structures with and without phosphatase treatment at 6 h post transfection (Figure 6E), phosphatase treatment led to substantial decrease in IFN-β secretion, particularly noticeable for CleanCap by secretion of IFN-β almost identical to untransfected MΦs (Figures 6E and 6F) and unexpected for this cap structure given the reported high incorporation efficacy (data provided by manufacturer).

Nucleotide modifications had a profound effect on cytokine secretion (Figure 7). Non-modified nucleotides persistently resulted in the highest level of TNF-α, IL-6, and IFN-β, similar to corresponding positive controls, both at low and high doses of IVT-mRNA (Figure 7). IVT-mRNA modified only with Ψ or 5meC resulted in significantly high levels of TNF-α and IL-6 secretion at 6 h and more drastically at 24 h upon transfection, both at low and high doses of mRNA when compared with untransfected MΦs (Figures 7A–7D). However, transfection-induced cytokine secretion was significantly reduced, but not abolished, when the combination of the two modifications, i.e., Ψ/5meC, was applied together (Figures 7A–7D). Uridine substitution with $me^1Ψ$ led to a significant reduction of TNF-α (Figures 7A and 7B) and IL-6 (Figures 7C and 7D) secretion at low dose, and high dose of IVT-mRNA, when compared with unmodified mRNA. In contrast, 5moU IVT-mRNA outperformed other modifications by completely preventing TNF-α and IL-6 induction, even at high doses of IVT-mRNA when measured at 6 h (Figures 7A and 7C) and 24 h (Figures 7B and 7D). Consistently, IFN-β secretion was found to be minimal for the 5moU modification (Figures 7E and 7F), whereas

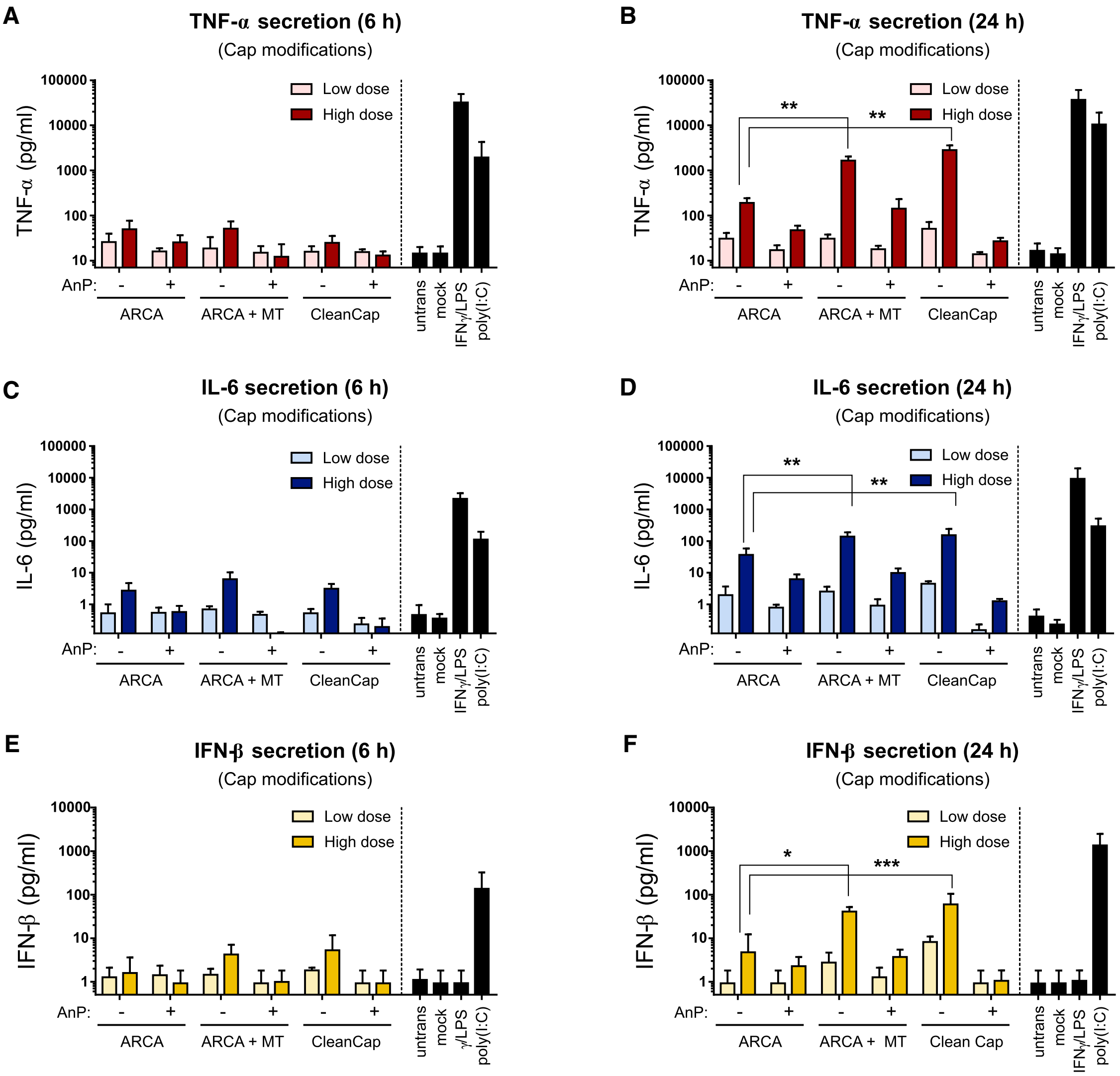

Figure 6. Cytokine secretion by macrophages transfected with cap modified IVT-mRNA

TNF-α secretion was measured (A) 6 h and (B) 24 h after transfection with low doses and high doses of IVT-mRNA. IL-6 secretion was quantified (C) 6 h and (D) 24 h post transfection. IFN-β secretion was evaluated (E) 6 h and (F) 24 h upon transfection. Mock transfection refers to carrier (i.e., LipoMM) without mRNA. Poly(I:C) was also transfected in low dose as positive control. For each condition, 125 $ng \cdot mL^{-1}$ and 500 $ng \cdot mL^{-1}$ of IVT-mRNA were used for transfection, referred here as low dose and high dose, respectively. Values are presented as mean ± SD. Error bars indicate SD of three independent experiments from three individual donors. Statistical differences are depicted with *$p < 0.05$, **$p < 0.005$, ***$p < 0.001$. Error bars indicate SD. ARCA, anti-reverse cap analog; MT, methyl-transferase; AnP, Antarctic phosphatase.

non-modified, Ψ-, 5meC-modified IVT-mRNA resulted in the highest level of antiviral response. While Ψ/5meC and me^{1}Ψ modifications were beneficial in reduction of IFN-β secretion both at low and high doses of IVT-mRNA at 6 h (Figure 7E), they failed to completely overcome the IFN-β production at high dose of mRNA when measured at 24 h. Overall, 5moU-modified IVT-mRNA was found to induce only minimal levels of cytokine secretion, in most cases similar to untransfected MΦs (Figure 7).

To ensure that the observed effects were not specific to the sequence of EGFP, an IVT-mRNA coding for another protein, i.e., mCherry, with a different nucleotide sequence was evaluated for four selected nucleotide modification conditions. The transfection efficiency, as well as level of mRNA expression revealed the same pattern of differences as observed for EGFP (Figure S7A). The immune activation was measured 24 h post transfection in terms of CD80 expression and IFN-β secretion. When plotted side-by-side to EGFP transfected macrophages, no remarkable difference was identified (Figures S7B and S7C). This result was consistent with dsRNA content of mCherry IVT-mRNA samples evaluated by dot blot (Figure S8).

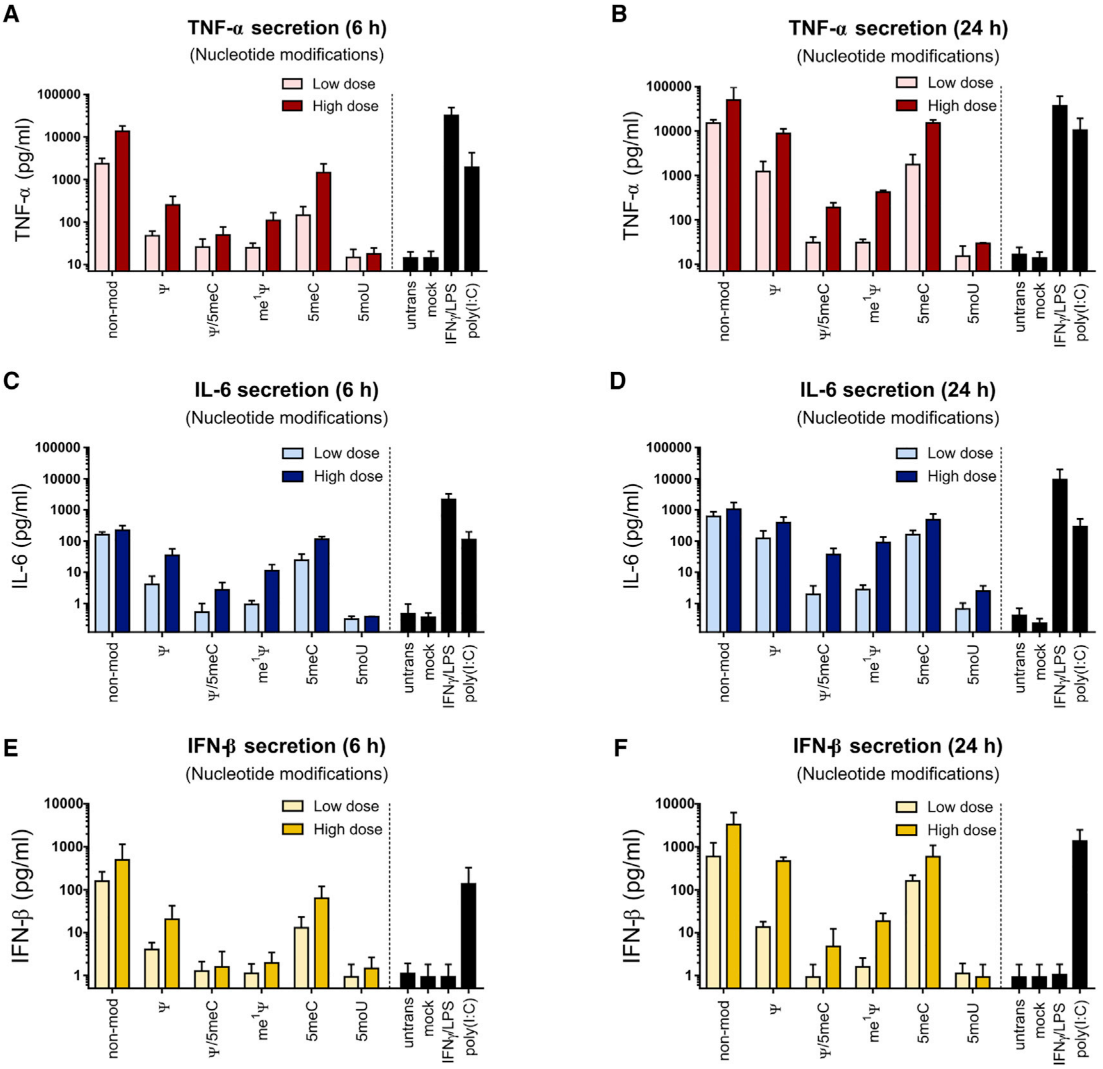

Figure 7. Cytokine secretion in macrophages transfected with IVT-mRNA with various nucleotide modifications

TNF-α secretion was measured (A) 6 h and (B) 24 h after transfection with low doses and high doses of different IVT-mRNA formula. IL-6 secretion was quantified (C) 6 h and (D) 24 h after transfection. IFN-β secretion was investigated (E) 6 h and (F) 24 h upon transfection. Mock transfection refers to carrier (i.e., LipoMM) without mRNA. Poly(I:C) was also transfected in low-dose positive control. For each condition, 125 $ng \cdot mL^{-1}$ and 500 $ng \cdot mL^{-1}$ of IVT-mRNA were used for transfection, referred here as low dose and high dose, respectively. Values are presented as mean ± SD. Error bars indicate SD of three independent experiments from three individual donors (n = 3). Ψ, pseudouridine; $me^1\Psi$, N^1-Methylpseudouridine; 5moU, 5-methoxy-uridine; 5meC, 5-methyl-cytidine.

Effect of HPLC purification on MΦ immune response triggered upon transfection of IVT-mRNA

While we have shown so far that immune responses due to delivery of synthesized mRNAs can be minimized if not abrogated by the proper choice of modified nucleotides, we also addressed the possibility of avoiding macrophage activation by an additional HPLC-purification step even for otherwise immunogenic IVT-mRNA chemistry, as previously described for other cell types.[31] The elimination of impurities and dsRNA by preparative chromatography, also proved by dot blot (Figure S8C), barely effected transfection efficiency and levels of transgene expression (Figures 8A–8E) in a series of experiments analogous to those presented in Figure 4. The only exception was the 2-fold increase in EGFP MFI for non-modified IVT-mRNA. By contrast, for IVT-mRNAs that were highly immunogenic in the unpurified stage, i.e., non-modified and Ψ-modified, cell activation measured both by CD80 levels (Figure 8F) and IFN-β production (Figures 8G and 8H) was largely reduced, confirming the efficacy of this technique in elimination of immune-stimulatory mRNA specimens and contaminants.

DISCUSSION

We investigated the effect of systematically varied IVT-mRNA chemistry, including various cap structures as well as nucleotide modifications in human monocyte-derived macrophages, analyzing transgene

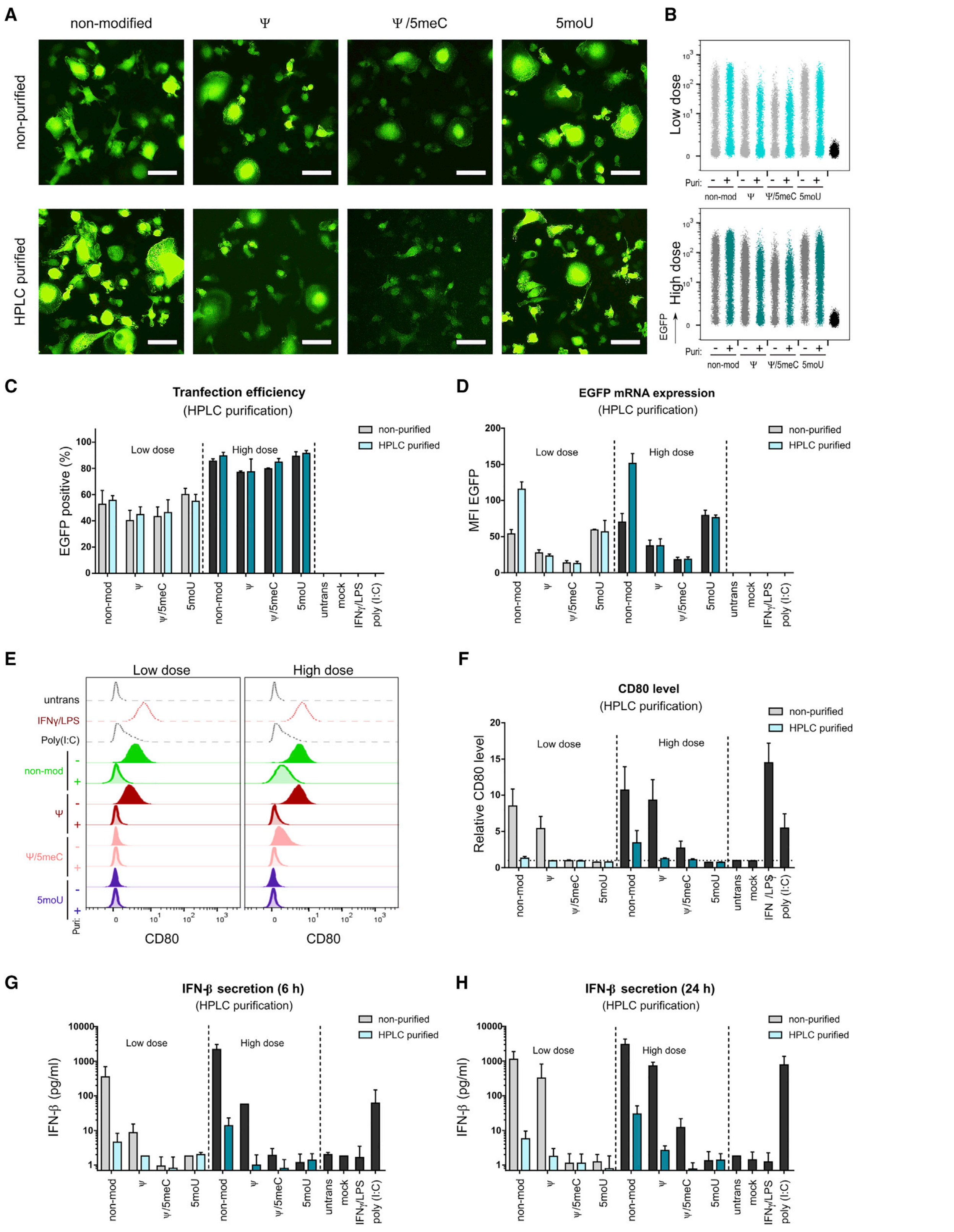

(legend on next page)

expression and activation of these primary cells. Being equipped with numerous sensors/receptors against a variety of pathogen-associated molecular patterns, macrophages play a pivotal role in innate immune response and can initiate adaptive immunity. Consequently, evaluation of their behavior is of high relevance for development of new therapeutics, such as mRNA vaccines. When compared with variations of cap structure, modification of nucleotides had the more pronounced effect on macrophages as identified by transgene expression levels and immunogenicity (Figure S9). While these biological responses could neither be attributed to differences in mRNA integrity nor potential unintended side-products of the T7-mediated *in vitro* transcription process, we found a partial correlation to the dsRNA content of transcripts, bringing up the necessity for extra purification steps. In fact, HPLC purification led to the reduction of dsRNA content and subsequently reduced the immunogenicity of the IVT-mRNA.

Given the sometimes striking effects of IVT-mRNA purification steps on both transgene expression and cell activation, this issue deserves further attention, as evidented by recent research. In line with our findings, Karikó et al. reported a direct correlation between dsRNA content of non-modified and Ψ-modified and Ψ/5meC-modified mRNA and type I IFN response of transfected dendritic cells.[31] However, elimination of dsRNA by HPLC alone was not sufficient for complete evasion of innate immune response, drawing attention to other aspects of IVT-mRNA, which could be involved in induction of immune activation.[29] Last, as a note of caution in considering a direct quantitative connection between dsRNA content and immune activation, we want to point out that the J2 antibody test used by us and some of the studies mentioned, to the best of our knowledge has not been rigorously validated to exclusively respond to dsRNA structures, irrespective of chemical modifications of the target transcripts.

Despite a high degree of similarity and functional equivalence, IVT-m RNA molecules can be distinguished from endogenous mRNA through differences in their chemical compositions and distinct trafficking routes. Therefore, many studies have been conducted to mimic intrinsic mRNA chemistry, such as the cap structure. Considering the fact that Cap 1 structure is more prevalent in higher eukaryotes than Cap 0,[42,43] in an *in vivo* study Vaidyanathan et al. investigated the immune stimulation and functional protein production by IVT-mRNA made of Cap 0 and Cap 1 structure, along with three other approaches, but did not find striking differences in their functionality and immunogenicity.[23] Likewise, we also found no remarkable differences in proinflammatory and antiviral responses in primary macrophages, as well as level of protein production between Cap 0- and Cap 1-modified IVT-mRNA. Our observation is also in agreement with a previous study,[43] where no differences in RIG-I-mediated immune activation was reported between ssRNA samples with Cap 0 versus a 2′-O-methylated cap, i.e., a Cap 1 structure. However, when cap modifications were examined and compared in dsRNA samples, Cap 1 was superior to Cap 0 in inhibition of RIG-I pathway.[43] In our experimental setting, a modest decrease of innate immune response with almost no detectable change in protein production level was observed for phosphatase-treated IVT-mRNAs. Notably, the dampening of immune response upon dephosphorylation was slightly more pronounced, when high doses of IVT-mRNA were applied.

In order to evaluate the impact of nucleotide chemical modifications on corresponding IVT-mRNA expression level and immune response, macrophages were transfected with low and high doses of IVT-mRNA in side-by-side experiments. Our results revealed that modification of IVT-mRNA with Ψ led to high level of protein production, but concurrently induced high levels of IFN-β, TNF-α, and IL-6 secretion. In line with these results, a previous study suggested an enhanced immunogenicity for Ψ-modified mRNA, which was correlated to dsRNA mediated-MDA5 stimulation.[16] The combination of Ψ and 5meC modifications, however, was effective in the reduction of IVT-mRNA immunogenicity. This finding is in agreement with previous reports by others[31] and us,[41] where we observed similar pattern throughout different doses, also when examined with different types of carriers such as polyplexes.[41]

Reduced protein expression observed for Ψ/5meC and 5meC-modified mRNA might be attributed to cell-autonomous immunity, which can be mediated by dsRNA interacting with PKR. Following activation by dsRNA or viral RNA, PKR monomers are phosphorylated and dimerize to form the active enzyme.[44] Dimerized PKR can

Figure 8. HPLC purification increases protein expression and ameliorates immune activation of macrophages

(A) Representative fluorescent images transfected with low dose of non-purified and HPLC-purified IVT-mRNAs comparing non-modified mRNA with Ψ-, Ψ/5meC-, and 5moU-modified mRNA. (B) Flow cytometric density plots indicating EGFP expression in macrophages transfected with low dose (top) and high dose (bottom) of IVT-mRNA comparing non-modified mRNA with Ψ-, Ψ/5meC-, and 5moU-modified and non-purified (gray color) versus HPLC-purified (blue color) conditions. (C) Transfection efficiency and (D) EGFP mRNA expression level quantified by flow cytometry and plotted as percentage and MFI of EGFP-positive cells, respectively. (E) Staggered histogram of CD80 levels in macrophages transfected with low doses (left) and high doses (right) of IVT-mRNA comparing non-modified mRNA with Ψ-, Ψ/5meC-, and 5moU-modified and non-purified with HPLC-purified samples. Untransfected, poly(I:C)-treated, and LPS/IFN-γ-activated macrophages served as negative, positive, and high control, respectively. (F) MFI of CD80 normalized to untransfected cells for macrophages transfected with low doses and high doses of non-purified versus HPLC-purified IVT-mRNAs comparing non-modified mRNA with Ψ-, Ψ/5meC-, and 5moU-modified IVT-mRNAs. Mock transfection refers to carrier (i.e., LipoMM) without mRNA. (G, H) IFN-β secretion after (G) 6 h and (H) 24 h upon transfection with low doses and high doses of non-purified versus HPLC-purified IVT-mRNAs comparing non-modified mRNA with Ψ-, Ψ/5meC-, and 5moU-modified IVT-mRNAs. Mock transfection refers to carrier (i.e., LipoMM) without mRNA. Untransfected, poly(I:C)-treated, and LPS/IFN-γ-activated macrophages served as negative, positive, and high control, respectively. For each condition, 125 $ng \cdot mL^{-1}$ and 500 $ng \cdot mL^{-1}$ of IVT-mRNA were used for transfection, referred here as low dose and high dose, respectively. Values are presented as mean ± SD. Error bars indicate SD of three independent experiments from three individual donors. Bar = 50 μm.

phosphorylate eIF2α, leading to translation inhibition. Consistently, Anderson et al. reported enhanced activation of PKR in a cell-free *in vitro* system for Ψ-modified IVT-mRNA compared with non-modified transcripts.[45] OAS can also be activated by dsRNA to polymerize ATP into oligomers of adenosine, which can specifically activate RNaseL that, in turn, mediates RNA degradation. OAS can be induced by type I IFNs. We found IFN-β production elevated by non-modified, Ψ-modified and 5meC-modified mRNA, but only for 5meC-modified mRNA a reduced mRNA expression was observed, indicating that PKR or OAS pathways could be activated by this modification. However, HPLC purification of 5meC-modified mRNA completely abolished the IFN-β secretion, while the EGFP expression remained at low level, indicating that interferon-induced cell-autologous mRNA decay pathways are not responsible for the reduced translation.[31]

Chemical modifications of uridine, including $me^1\Psi$, and 5moU outperformed others in terms of augmenting mRNA expression level, as well as substantially reducing both antiviral and proinflammatory cytokine secretion. In particular, 5moU-modified IVT-mRNA led to almost complete evasion of IFN-β secretion, a result that even extended to high-dose IVT-mRNA transfection. This finding is in line with an earlier report, where in an *in vivo* experimental setup a similar pattern of reduced activation was reported for 5moU, when compared with unmodified and other uridine and cytidine chemical modifications.[23] Another study by Nelson and colleagues also suggested that $me^1\Psi$ modification of IVT-mRNA reduced, but not eliminated the expression of inflammatory chemokine, CXCL-10, produced by transfected primary human monocyte-derived macrophages. However, when this modification was combined with an extra purification step using reversed-phase high-performance liquid chromatography (RP-HPLC), it resulted in the pronounced inhibition of innate immune response to background level, examined both *in vitro* and *in vivo*.[29] Both of the recently developed mRNA-based SARS-CoV-2 vaccines that are on the market as of the beginning of 2021 rely on $me^1\Psi$-modified IVT-mRNA formulated in lipid-based nanoparticles,[10,46] where the moderate activation of the immune system is often intended. However, our study demonstrates that, at least for macrophages, the immune reaction can be reduced almost to background levels without employing sophisticated extra purification steps, only by using 5moU modification of IVT-mRNA, thus facilitating potential applications that require minimal immune stimulation.

We observed that nucleotide modifications of IVT-mRNA with $me^1\Psi$ and 5moU increased yield of protein production. This was in line with a previous report that attributed the enhanced expression level of $me^1\Psi$-modified IVT-mRNA to increased ribosome loading density and higher ribosomal recycling rate compared with mRNA containing canonical uridine in cell-free translation systems.[47] Other studies, however, attribute high mRNA expression to reduced inhibitory effects of cell-autonomous antiviral defense mechanism mediated by PKR or OAS leading to mRNA decay or translation inhibition.[16] Noteworthy, we found that the pattern in immune activation observed between the nucleotide modifications tested were not dose-sensitive. However, variations of protein synthesis were more obvious upon transfecting low IVT-mRNA doses and deviation of immune response at high doses of IVT-mRNA.

Elucidation of the mechanisms involved in modulation of immune response by chemical modification of nucleotides, in particular uridine, were the subject of previous studies that can be summarized as follows: (1) transcripts containing modified nucleotides were reported to have less binding affinity to endosomal ssRNA sensors TLR7/8[25,27,48]; (2) several studies reported that the incorporation of modified nucleotides in transcripts results in less mRNA sensor activation as shown for cytosolic, RIG-I,[43] MDA5,[16] and TLR-3 [18,27], with subsequent reduction of type I IFN and proinflammatory cytokines secretion, as well as reduction of PKR activity resulting in increased translation[24]; (3) nucleotide modifications can reduce unspecific promoter-independent activities of T7 RNA polymerase during the IVT process, like synthesis of long dsRNA by-products[16]; and last, (4) reduction of dsRNA potency in receptor stimulation.[49]

Conclusion

In the present study, we investigated the effects of different cap and nucleotide modifications of IVT-mRNA upon macrophage transfection. The different modifications and/or treatments of the transcripts' 5′-end had only modest consequences for protein expression and macrophage activation. Intriguingly, the use of nucleotide modifications had a major impact on the overall biological performance of the IVT-mRNAs. Chemical modification of uridine, in particular 5moU showed the highest levels of protein production with negligible induction of inflammatory macrophage responses. While most envisaged therapeutic applications of IVT-mRNA will profit from the highest possible protein yield per transcript delivered, such applications, ranging from protein replacement, expression of tumor antigens, or "classical" vaccination strategies, have distinct requirements for eliciting inflammatory responses, both quantitatively and qualitatively. Thus, while further experiments are required to elucidate molecular mechanisms corresponding to each specific modification, this study should motivate consideration of human macrophages as a mediator of custom-tailored mRNA-induced inflammatory reactions for the intended therapeutic application.

MATERIALS AND METHODS

IVT-mRNA synthesis with various chemical modifications

Synthesis of mRNA was performed via *in vitro* transcription by T7 RNA polymerase in two groups to introduce chemical modifications to cap structures and nucleotides described in detail as follows; also see Figure 2, and Table S1 for more information about chemical composition and overview of synthesis process.

1) Template linearization

The plasmid DNA (pDNA) vector, pRNA2-$(A)_{128}$[50] was utilized as template for synthesis of mRNAs with ARCA as cap structure. It consists of a standard T7 promoter, a short 5′-UTR containing a Kozak

sequence, an EGFP coding region, and a head-to-tail duplicated human β-globin 3′-UTR followed by a 128-base-pair (bp) polyadenine [poly(A)] sequence.

A modified version of this plasmid comprised the features (mentioned above) aside from an altered T7 promoter transcriptional start site by changing from "GG" to "AG". In this way, the standard promoter sequence 5′-TAATACGACTCACTATAGG-3′ is changed to 5′-TAATACGACTCACTATAAG-3′, in order to accommodate integration of CleanCap AG as cap structure.

pDNA templates were linearized with BspMI restriction enzyme (New England Biolabs, Frankfurt, Germany), and purified by adding 0.05 volume of 3 M sodium acetate (Thermo Fisher Scientific, Darmstadt, Germany), 0.1 volume of 0.5 M EDTA (Thermo Fisher Scientific) and 2 volumes 100% EtOH (Carl Roth, Karlsruhe, Germany). Upon incubation at −20°C for 1 h, samples were centrifuged at 14,000 × *g* at 4°C for 30 min. The resulting DNA pellets were air-dried and resuspended in UltraPure nuclease-free sterile water (Merck Millipore, Darmstadt, Germany) for downstream experiments.

2) T7-mediated *in vitro* transcription

mRNAs were synthesized using TranscriptAid T7 High Yield Transcription Kit (Thermo Fisher Scientific) according to the manufacturer's instructions. Cap modified mRNAs were synthesized by co-transcriptional incorporation of either ARCA (Jena Bioscience, Jena, Germany) as a dinucleotide Cap 0, or CleanCap AG (TriLink, San Diego, CA) as a trinucleotide Cap 1 analog, at final concentrations of 5 mM. In both cases, IVT-mRNAs were also chemically modified by complete substitution of uridine and cytidine with pseudouridine (Ψ) (Jena Bioscience) and 5-methyl-cytidine (5meC) (Jena Bioscience).

mRNAs with chemical modification of nucleotides were synthesized with complete substitution of either uridine or cytidine with corresponding modified nucleotides. Hereby, uridine is fully substituted either by Ψ, N1-methyl-pseudouridine ($me^1Ψ$) (Jena Bioscience) or 5-methoxy-uridine (5moU) (Jena Bioscience); cytidine is fully substituted by 5meC. In addition, in one case, uridine and cytidine were modified by supplying a combination of Ψ and 5meC. All nucleotides were used at final concentration of 5 mM in the transcription reaction, except GTP, which was decreased to 1.5 mM to increase capping efficiency. The 5′-end of these IVT-mRNAs was determined co-transcriptionally by ARCA incorporation. IVT-mRNAs were purified using lithium chloride and resuspended in UltraPure nuclease-free sterile water supplemented with 0.1 mM EDTA.

3) Cap methylation

In order to generate Cap 1 from ARCA-capped mRNA, IVT products were purified with RNeasy kit (Qiagen, Hilden, Germany), denatured at 65°C for 5 min, treated with mRNA Cap 2′-O-methyl-transferase (5 U/μg) (M0366S) (New England Biolabs) in 1x capping buffer and 0.2 mM S-adenosylmethionine (SAM) for 1 h at 37°C, and then repurified by using a RNeasy kit.

4) 5′-end dephosphorylation

Dephosphorylation of the 5′-end of potentially uncapped IVT-mRNAs was performed by treatment with 1 U/μg Antarctic phosphatase (M0289L) (New England Biolabs) in 1x Antarctic phosphatase buffer. All transcripts were purified from reaction mixture by overnight incubation at −20°C in lithium chloride solution (Thermo Fisher Scientific) at final concentration of 2.8 M. Upon centrifugation at 14,000 × *g* at 4°C for 30 min, IVT-mRNA products were washed with 70% EtOH, and the air-dried pellets were then resuspended in UltraPure nuclease-free sterile water containing 0.1 mM EDTA.

IVT-mRNA purification with HPLC

Four of the mRNAs with different nucleotide chemistries were purified by HPLC using 7.8 × 50 mm alkylated non-porous polystyrene-divinylbenzene (PS-DVB)-based RNASep Prep RNA purification column (ADS Biotech, Hillington Park, Glasgow). WAVE Optimized Buffer A contained 0.1 M triethylammonium acetate in water (ADS Biotech), and WAVE Optimized Buffer B, composed of 0.1 M TEAA in 25% Acetonitrile (ADS Biotech), were used as the buffer system throughout. The purification was done according to the previously published protocol.[31] The collected fractions were desalted via Amicon Ultra-15 centrifugal filter unit (30 K membrane) (Merck Millipore), and the mRNA samples were subsequently recovered from fractions using overnight precipitation by 1:10 vol NaOAc and 1 vol isopropanol and glycogen (Roche).

IVT-mRNA characterization

The concentrations of IVT-mRNAs were determined using UV/Vis-spectroscopy (NanoDrop 1000 Spectrophotometer; Peqlab, Erlangen, Germany) and integrity of transcripts was analyzed by denaturing agarose gel electrophoresis.

Measurement of dsRNA by dot blot assay

The dsRNA content of synthesized IVT-mRNAs was analyzed by dot plot assay according to the previously published protocol in Baiersdörfer et al.[32] Briefly, IVT-mRNA samples were blotted on a supercharged nylon membrane (GE Healthcare Life Science, Freiburg, Germany) using a 96-well bio-dot silicon gasket (Bio-Rad, Munich, Germany), at concentration of 1,000 ng per dot. In parallel, 1:4 serial dilutions of 142-bp dsRNA positive control (Jena Bioscience), were blotted on the same membrane, starting at 1,000 ng as the highest concentration. Besides, 1,000 ng of single-stranded polyadenylic acid (poly(A)) (Sigma-Aldrich, Hamburg, Germany) was blotted side-by-side as negative control. After loading of the samples, the membrane was air-dried and blocked in 5% (w/v) blotting grade non-fat dry milk (Bio-Rad) in 1x Tris-buffered saline with Tween 20 (TBS-T) buffer (Cell Signaling Technology, Leiden, Netherlands) at room temperature (RT) for 1 h. The membrane was incubated with 15 mL 1:5,000 diluted dsRNA-specific monoclonal antibody

(mAb) J2 (English & Scientific Consulting, Szirák, Hungary) in 1% (w/v) blotting grade non-fat dried milk at 4°C overnight on a rocker shaker. The membrane was washed three times with 30 mL 1x TBS-T, and incubated with 15 mL 1:2,500 diluted horseradish peroxidase (HRP)-conjugated goat anti-mouse immunoglobulin G (IgG) (Thermo Fisher Scientific) in 1% (w/v) blotting grade non-fat dry milk at RT for 1 h. Upon washing three times with 30 mL 1x TBS-T, the membrane was treated with 0.1 mL/cm enhanced chemiluminescence (ECL) western blotting detection reagent (GE Healthcare Life Science) in the dark and immediately analyzed using a ChemiDoc MP imaging system (Bio-Rad). For comparison of different samples, signal intensities were measured by corresponding densitometry software, Image Lab (Bio-Rad), using volume tools. The dsRNA content of samples was interpolated using dsRNA positive control standard curve, then normalized to the total amount of mRNA loaded per dot, i.e., 1,000 ng, and eventually presented as dsRNA/total mRNA % (w/w).

In vitro culture of primary human macrophages

Primary human macrophages were differentiated from monocytes, according to the protocol reported previously.[41] In brief, monocytes were purified from buffy coat-derived peripheral blood mononuclear cells (Deutsches Rotes Kreuz, Berlin, Germany; ethics vote EA2/018/16; Charité University Medicine Berlin, Berlin, Germany), by magnetic sorting using the Monocyte Isolation Kit II (Miltenyi Biotec, Bergisch Gladbach, Germany) according to the manufacturer's instruction. CD14-positive monocytes were subsequently cultured in very low endotoxin (VLE) RPMI 1640 (PAN-Biotech, Aidenbach, Germany), supplemented with 10% (v/v) fetal bovine serum (FBS) (Sigma-Aldrich), and 50 ng/mL human macrophage colony stimulating factor (Miltenyi Biotec) at 37°C in an atmosphere with 5 vol % CO_2 for 7 days, with medium change at day 3. Upon differentiation, macrophages were cultured in VLE RPMI, only supplemented with 10% (v/v) FBS for subsequent experiments.

IVT-mRNA transfection

The transfection experiment was performed by complexing IVT-mRNA with Lipofectamine MessengerMAX (LipoMM) (Thermo Fisher Scientific) reagent as follows: LipoMM reagent was diluted in Opti-MEM reduced serum medium (Thermo Fisher Scientific) at a 1:50 volume ratio, and incubated for 10 min at RT. The resulting solution was then mixed with equal volume of Opti-MEM containing 4 ng/μL of IVT-mRNA. Complexed IVT-mRNAs were briefly vortexed and incubated for 10 min at RT. The corresponding volumes to deliver 125 ng and 500 ng IVT-mRNA per 1 mL of cell culture medium were added drop-wise to each well, referred to as low dose and high dose, respectively, throughout all experiments. Macrophages were transfected with poly(I:C) (125 ng/mL), mimicking dsRNA as positive control. Besides, transfection reagent mixed with Opti-MEM without addition of IVT-mRNA was considered as mock negative control. Untransfected cells treated with 2 μg/mL LPS (Enzo Life Sciences) and 10 ng/mL IFN-γ (Miltenyi Biotec) served as positive control for innate immune response. Macrophages were imaged with inverted microscope ELIPSE T*i*-U equipped pE-300lite LED light source (Nikon, Düsseldorf, Germany) 24 h after transfection, and images were analyzed with NIS-Elements imaging software, version 4.51 (Nikon). Of note, to avoid unintended cell activation due to potential endotoxin contamination, all reagents used for IVT-mRNA synthesis as well as cell isolation and cell culture mediums were purchased as very low endotoxin and/or endotoxin-free grade. Besides, IVT-mRNA batches were regularly tested for endotoxin level using EndoLISA assay according to guidance for industry pyrogen and endotoxins testing from the Food and Drug Administration (FDA), and consistently proved to be endotoxin-free (EU < 0.05).

Evaluation of IVT-mRNA expression and CD80 expression with flow cytometry

At 24 h post transfection, macrophages were harvested by scraping, then washed with cold autoMACS running buffer (Miltenyi Biotec). Cells were incubated with FcR blocking reagent (Miltenyi Biotec) for 10 min at 4°C, to avoid unspecific antibody binding. Upon washing, cells were stained with CD80-PE (clone L307.4) (BD Bioscience, Heidelberg, Germany) antibody with dilution factor 1:100 (5 μg/mL final concentration) at 4°C for 10 min. After a final washing step, cells were analyzed with MACSQuant VYB (Miltenyi Biotec). For live-dead discrimination, DAPI, at a final concentration of 1 μg/mL, was added to each sample immediately before measurement. All flow cytometric data were analyzed by FlowJo software V10 using the previously established gating strategy.[41] Briefly, cells were initially identified from debris by gating on forward versus side scatter area (FSC-A versus SSC-A) dot plots, followed by exclusion of aggregated cells using forward scatter area against height (FSC-A versus FSC-H). DAPI-negative cells were identified as live cells. EGFP-positive cell populations were determined among live single cells, by gating with respect to untransfected negative controls.

Cytokine measurements

Macrophage culture media were collected at 6 h and 24 h post transfection, centrifuged at 1,000 × *g* at 4°C for 15 min to remove possible cell debris, and the supernatants were preserved at −20°C until downstream measurements. Concentrations of cytokines were measured by Bio-Plex immunoassay (Bio-Rad) using Bio-Plex standards including Pro Human Cytokine Screening Group 1 (171D50001; Bio-Rad) for TNF-α and IL-6, and Pro Human Inflammation Panel 1 (171DL0001) (Bio-Rad) for IFN-β, according to corresponding manufacturers protocol. Briefly, 50 μL of 1x magnetic beads conjugated with capture antibody were added to each well of the 96-well assay plate, and washed twice with 1x wash buffer (Bio-Rad) in a Bio-Plex Pro II wash station (Bio-Rad). Subsequently, 50 μL of a dilution series of reconstituted cytokine standards and sample supernatants were added to prewashed beads and incubated on a shaker at 900 rpm at RT for either 30 min or 1 h, depending on the assay type. The plates were washed three times with 1x wash buffer, and then incubated with 1x biotinylated detection antibodies on a shaker at 900 rpm at RT for 30 min. Upon three times washing, 1x PE-conjugated streptavidin was added to each well and plates were incubated at 900 rpm at RT for 10 min. After three last washing steps,

beads were resuspended in 125 μL assay buffer, shaken at 900 rpm for 30 s and measured by Bio-Plex 200 System (Bio-Rad).

Statistics and software

Data were statistically analyzed using Prism 7.00 software (GraphPad, San Diego, CA). All data are presented as means ± standard deviation (SD) of at least three independent experiments. two-way ANOVA test was performed for multiple comparisons between different groups with a 95% confidence interval. Statistical significance was considered as $p < 0.05$.

Figures 1 and 2 created with BioRender.com.

SUPPLEMENTAL INFORMATION

Supplemental information can be found online at https://doi.org/10.1016/j.omtn.2022.01.004.

ACKNOWLEDGMENTS

This work was financially supported by the Helmholtz Association through program-oriented funding and through Helmholtz Graduate School of Macromolecular Bioscience (MacroBio, VH-GS-503), as well as the German Federal Ministry of Education and Research through the Program Health Research (Grant No. 13GW0098). The authors acknowledge Dr. Irena Vlatkovic (BioNTech RNA Pharmaceuticals GmbH) for providing detailed protocol of dot blot assay, and Marko Schwestka for his valuable technical support.

AUTHOR CONTRIBUTIONS

M.G., H.M., and T.R. conceptualized the study; M.G., H.M., and T.R. developed the methodology; H.M., and L.A. performed study investigation; H.M. wrote the original draft; M.G., T.R., and A.L. reviewed and edited the manuscript; M.G. and A.L. acquired the funding; M.G. and A.L. acquired the resources; M.G. and A.L. supervised the study.

DECLARATION OF INTERESTS

The authors declare no competing interests.

REFERENCES

1. Sahin, U., Karikó, K., and Türeci, Ö. (2014). mRNA-based therapeutics–developing a new class of drugs. Nat. Rev. Drug Discov. *13*, 759–780.
2. Sergeeva, O.V., Koteliansky, V.E., and Zatsepin, T.S. (2016). mRNA-based therapeutics - advances and perspectives. Biochemistry *81*, 709–722.
3. Coutinho, M.F., Matos, L., Santos, J.I., and Alves, S. (2019). RNA therapeutics: how far have we gone? Adv. Exp. Med. Biol. *1157*, 133–177.
4. Weissman, D. (2015). mRNA transcript therapy. Expert Rev. Vaccin. *14*, 265–281.
5. Meng, Z., O'Keeffe-Ahern, J., Lyu, J., Pierucci, L., Zhou, D., and Wang, W. (2017). A new developing class of gene delivery: messenger RNA-based therapeutics. Biomater. Sci. *5*, 2381–2392.
6. Magadum, A., Kaur, K., and Zangi, L. (2019). mRNA-based protein replacement therapy for the heart. Mol. Ther. *27*, 785–793.
7. Pardi, N., Hogan, M.J., Porter, F.W., and Weissman, D. (2018). mRNA vaccines — a new era in vaccinology. Nat. Rev. Drug Discov. *17*, 261–279.
8. Linares-Fernández, S., Lacroix, C., Exposito, J.Y., and Verrier, B. (2020). Tailoring mRNA vaccine to balance innate/adaptive immune response. Trends Mol. Med. *26*, 311–323.
9. Reinhard, K., Rengstl, B., Oehm, P., Michel, K., Billmeier, A., Hayduk, N., Klein, O., Kuna, K., Ouchan, Y., Wöll, S., et al. (2020). An RNA vaccine drives expansion and efficacy of claudin-CAR-T cells against solid tumors. Science *367*, 446–453.
10. Corbett, K.S., Edwards, D.K., Leist, S.R., Abiona, O.M., Boyoglu-Barnum, S., Gillespie, R.A., Himansu, S., Schäfer, A., Ziwawo, C.T., DiPiazza, A.T., et al. (2020). SARS-CoV-2 mRNA vaccine design enabled by prototype pathogen preparedness. Nature *586*, 567–571.
11. Moradian, H., Lendlein, A., and Gossen, M. (2020). Strategies for simultaneous and successive delivery of RNA. J. Mol. Med. (Berl) *98*, 1767–1779.
12. Mulligan, M.J., Lyke, K.E., Kitchin, N., Absalon, J., Gurtman, A., Lockhart, S., Neuzil, K., Raabe, V., Bailey, R., Swanson, K.A., et al. (2020). Phase I/II study of COVID-19 RNA vaccine BNT162b1 in adults. Nature *586*, 589–593.
13. Blanchard, E.L., Loomis, K.H., Bhosle, S.M., Vanover, D., Baumhof, P., Pitard, B., Zurla, C., and Santangelo, P.J. (2019). Proximity ligation assays for in situ detection of innate immune activation: focus on in vitro-transcribed mRNA. Mol. Ther. Nucleic Acids *14*, 52–66.
14. Kawai, T., and Akira, S. (2008). Toll-like receptor and RIG-1-like receptor signaling. Ann. New York Acad. Sci. *1143*, 1–20.
15. Züst, R., Cervantes-Barragan, L., Habjan, M., Maier, R., Neuman, B.W., Ziebuhr, J., Szretter, K.J., Baker, S.C., Barchet, W., Diamond, M.S., et al. (2011). Ribose 2'-O-methylation provides a molecular signature for the distinction of self and non-self mRNA dependent on the RNA sensor Mda5. Nat. Immunol. *12*, 137–143.
16. Mu, X., Greenwald, E., Ahmad, S., and Hur, S. (2018). An origin of the immunogenicity of in vitro transcribed RNA. Nucleic Acids Res. *46*, 5239–5249.
17. Devoldere, J., Dewitte, H., De Smedt, S.C., and Remaut, K. (2016). Evading innate immunity in nonviral mRNA delivery: don't shoot the messenger. Drug Discov. Today *21*, 11–25.
18. Akira, S., and Takeda, K. (2004). Toll-like receptor signalling. Nat. Rev. Immunol. *4*, 499–511.
19. Hornung, V., Ellegast, J., Kim, S., Brzózka, K., Jung, A., Kato, H., Poeck, H., Akira, S., Conzelmann, K.K., Schlee, M., et al. (2006). 5'-Triphosphate RNA is the ligand for RIG-I. Science *314*, 994–997.
20. Macmicking, J.D. (2012). Interferon-inducible effector mechanisms in cell-autonomous immunity. Nat. Rev. Immunol. *12*, 367–382.
21. Lemaire, P.A., Anderson, E., Lary, J., and Cole, J.L. (2008). Mechanism of PKR activation by dsRNA. J. Mol. Biol. *381*, 351–360.
22. Eley, H.L., Russell, S.T., and Tisdale, M.J. (2010). Mechanism of activation of dsRNA-dependent protein kinase (PKR) in muscle atrophy. Cell Signal. *22*, 783–790.
23. Vaidyanathan, S., Azizian, K.T., Haque, A., Henderson, J.M., Hendel, A., Shore, S., Antony, J.S., Hogrefe, R.I., Kormann, M.S.D., Porteus, M.H., et al. (2018). Uridine depletion and chemical modification increase Cas9 mRNA activity and reduce immunogenicity without HPLC purification. Mol. Ther. Nucleic Acids *12*, 530–542.
24. Nallagatla, S.R., and Bevilacqua, P.C. (2008). Nucleoside modifications modulate activation of the protein kinase PKR in an RNA structure-specific manner. RNA *14*, 1201–1213.
25. Karikó, K., Buckstein, M., Ni, H., and Weissman, D. (2005). Suppression of RNA recognition by Toll-like receptors: the impact of nucleoside modification and the evolutionary origin of RNA. Immunity *23*, 165–175.
26. Karikó, K., Muramatsu, H., Welsh, F.A., Ludwig, J., Kato, H., Akira, S., and Weissman, D. (2008). Incorporation of pseudouridine into mRNA yields superior nonimmunogenic vector with increased translational capacity and biological stability. Mol. Ther. *16*, 1833–1840.
27. Kormann, M.S., Hasenpusch, G., Aneja, M.K., Nica, G., Flemmer, A.W., Herber-Jonat, S., Huppmann, M., Mays, L.E., Illenyi, M., Schams, A., et al. (2011). Expression of therapeutic proteins after delivery of chemically modified mRNA in mice. Nat. Biotechnol. *29*, 154–157.
28. Feyrer, H., Munteanu, R., Baronti, L., and Petzold, K. (2020). One-pot production of RNA in high yield and purity through cleaving tandem transcripts. Molecules *25*, 1142.

29. Nelson, J., Sorensen, E.W., Mintri, S., Rabideau, A.E., Zheng, W., Besin, G., Khatwani, N., Su, S.V., Miracco, E.J., Issa, W.J., et al. (2020). Impact of mRNA chemistry and manufacturing process on innate immune activation. Sci. Adv. *6*, eaaz6893.

30. Wu, M.Z., Asahara, H., Tzertzinis, G., and Roy, B. (2020). Synthesis of low immunogenicity RNA with high-temperature in vitro transcription. RNA *26*, 345–360.

31. Karikó, K., Muramatsu, H., Ludwig, J., and Weissman, D. (2011). Generating the optimal mRNA for therapy: HPLC purification eliminates immune activation and improves translation of nucleoside-modified, protein-encoding mRNA. Nucleic Acids Res. *39*, e142.

32. Baiersdörfer, M., Boros, G., Muramatsu, H., Mahiny, A., Vlatkovic, I., Sahin, U., and Karikó, K. (2019). A facile method for the removal of dsRNA contaminant from in vitro-transcribed mRNA. Mol. Ther. Nucleic Acids *15*, 26–35.

33. Xu, Y., Huang, L., Kirschman, J.L., Vanover, D.A., Tiwari, P.M., Santangelo, P.J., Shen, X., and Russell, D.G. (2019). Exploitation of synthetic mRNA to drive immune effector cell recruitment and functional reprogramming in vivo. J. Immunol. *202*, 608–617.

34. Ponzoni, M., Pastorino, F., Di Paolo, D., Perri, P., and Brignole, C. (2018). Targeting macrophages as a potential therapeutic intervention: impact on inflammatory diseases and cancer. Int. J. Mol. Sci. *19*, 1953.

35. Navegantes, K.C., de Souza Gomes, R., Pereira, P.A.T., Czaikoski, P.G., Azevedo, C.H.M., and Monteiro, M.C. (2017). Immune modulation of some autoimmune diseases: the critical role of macrophages and neutrophils in the innate and adaptive immunity. J. Transl. Med. *15*, 36.

36. Ackermann, M., Dragon, A.C., and Lachmann, N. (2020). The immune-modulatory properties of iPSC-derived antigen-presenting cells. Transfus. Med. Hemother *47*, 444–453.

37. Guerriero, J.L. (2019). Macrophages: their untold story in T cell activation and function. Int. Rev. Cell Mol. Biol. *342*, 73–93.

38. Merkley, S.D., Chock, C.J., Yang, X.O., Harris, J., and Castillo, E.F. (2018). Modulating T cell responses via autophagy: the intrinsic influence controlling the function of both antigen-presenting cells and T cells. Front. Immunol. *9*, 2914.

39. Hume, D.A. (2008). Macrophages as APC and the dendritic cell myth. J. Immunol. *181*, 5829.

40. Scharenberg, S.G., Poletto, E., Lucot, K.L., Colella, P., Sheikali, A., Montine, T.J., Porteus, M.H., and Gomez-Ospina, N. (2020). Engineering monocyte/macrophage−specific glucocerebrosidase expression in human hematopoietic stem cells using genome editing. Nat. Commun. *11*, 3327.

41. Moradian, H., Roch, T., Lendlein, A., and Gossen, M. (2020). mRNA transfection-induced activation of primary human monocytes and macrophages: dependence on carrier system and nucleotide modification. Sci. Rep. *10*, 4181.

42. Schuberth-Wagner, C., Ludwig, J., Bruder, A.K., Herzner, A.M., Zillinger, T., Goldeck, M., Schmidt, T., Schmid-Burgk, J.L., Kerber, R., Wolter, S., et al. (2015). A conserved histidine in the RNA sensor RIG-I controls immune tolerance to N1-2'O-methylated self RNA. Immunity *43*, 41–51.

43. Devarkar, S.C., Wang, C., Miller, M.T., Ramanathan, A., Jiang, F., Khan, A.G., Patel, S.S., and Marcotrigiano, J. (2016). Structural basis for m7G recognition and 2'-O-methyl discrimination in capped RNAs by the innate immune receptor RIG-I. Proc. Natl. Acad. Sci. U S A. *113*, 596–601.

44. Nanduri, S., Carpick, B.W., Yang, Y., Williams, B.R.G., and Qin, J. (1998). Structure of the double-stranded RNA-binding domain of the protein kinase PKR reveals the molecular basis of its dsRNA-mediated activation. EMBO J. *17*, 5458–5465.

45. Anderson, B.R., Muramatsu, H., Nallagatla, S.R., Bevilacqua, P.C., Sansing, L.H., Weissman, D., and Karikó, K. (2010). Incorporation of pseudouridine into mRNA enhances translation by diminishing PKR activation. Nucleic Acids Res. *38*, 5884–5892.

46. Sahin, U., Muik, A., Derhovanessian, E., Vogler, I., Kranz, L.M., Vormehr, M., Baum, A., Pascal, K., Quandt, J., Maurus, D., et al. (2020). COVID-19 vaccine BNT162b1 elicits human antibody and TH1 T cell responses. Nature *586*, 594–599.

47. Svitkin, Y.V., Cheng, Y.M., Chakraborty, T., Presnyak, V., John, M., and Sonenberg, N. (2017). N1-methyl-pseudouridine in mRNA enhances translation through eIF2α-dependent and independent mechanisms by increasing ribosome density. Nucleic Acids Res. *45*, 6023–6036.

48. Freund, I., Eigenbrod, T., Helm, M., and Dalpke, A.H. (2019). RNA modifications modulate activation of innate toll-like receptors. Genes *10*, 92.

49. Durbin, A.F., Wang, C., Marcotrigiano, J., and Gehrke, L. (2016). RNAs containing modified nucleotides fail to trigger RIG-I conformational changes for innate immune signaling. mBio *7:e00833-16*.

50. Williams, D.J., Puhl, H.L., and Ikeda, S.R. (2010). A simple, highly efficient method for heterologous expression in mammalian primary neurons using cationic lipid-mediated mRNA transfection. Front. Neurosci. *4*, 181.

OMTN, Volume *27*

Supplemental information

Chemical modification of uridine modulates mRNA-mediated proinflammatory and antiviral response in primary human macrophages

Hanieh Moradian, Toralf Roch, Larissa Anthofer, Andreas Lendlein, and Manfred Gossen

Supporting Information for

Chemical modification of uridine modulates mRNA-mediated proinflammatory and antiviral response in primary human macrophages

Hanieh Moradian[1, 2, 3], Toralf Roch[4, 5,6], Larissa Anthofer[1, 2], Andreas Lendlein[1, 2, 3], Manfred Gossen[1, 2, 1]

1 Institute of Active Polymers, Helmholtz-Zentrum Hereon, Kantstr. 55, 14513 Teltow, Germany
2 Berlin-Brandenburg Center for Regenerative Therapies (BCRT), Föhrerstr. 15, 13353 Berlin, Germany
3 Institute of Biochemistry and Biology, University of Potsdam, Karl-Liebknecht-Str. 24-25, 14476 Potsdam, Germany
4 Berlin Institute of Health at Charité – Universitätsmedizin Berlin, BIH Center for Regenerative Therapies (BCRT), Charitéplatz 1, 10117 Berlin, Germany
5 Charité – Universitätsmedizin Berlin, Corporate Member of Freie Universität Berlin, Humboldt-Universität zu Berlin, Berlin Center for Advanced Therapies, Augustenburger Platz 1, 13353 Berlin, Germany
6 Center for Translational Medicine, Immunology, and Transplantation, Medical Department I, Marien Hospital Herne, University Hospital of the Ruhr-University Bochum, Hölkeskampring 40, 44625 Herne, Germany

* Corresponding Author, email address: manfred.gossen@hereon.de

[1] Corresponding author: Dr. Manfred Gossen, Institute of Active Polymers and Berlin-Brandenburg Center for Regenerative Therapies, Helmholtz-Zentrum Hereon, Kantstr. 55, 14513 Teltow, Germany. Tel.: +49 (0) 30 450539-491; Fax: +49 (0)30 450539-991; E-mail: manfred.gossen@hereon.de.

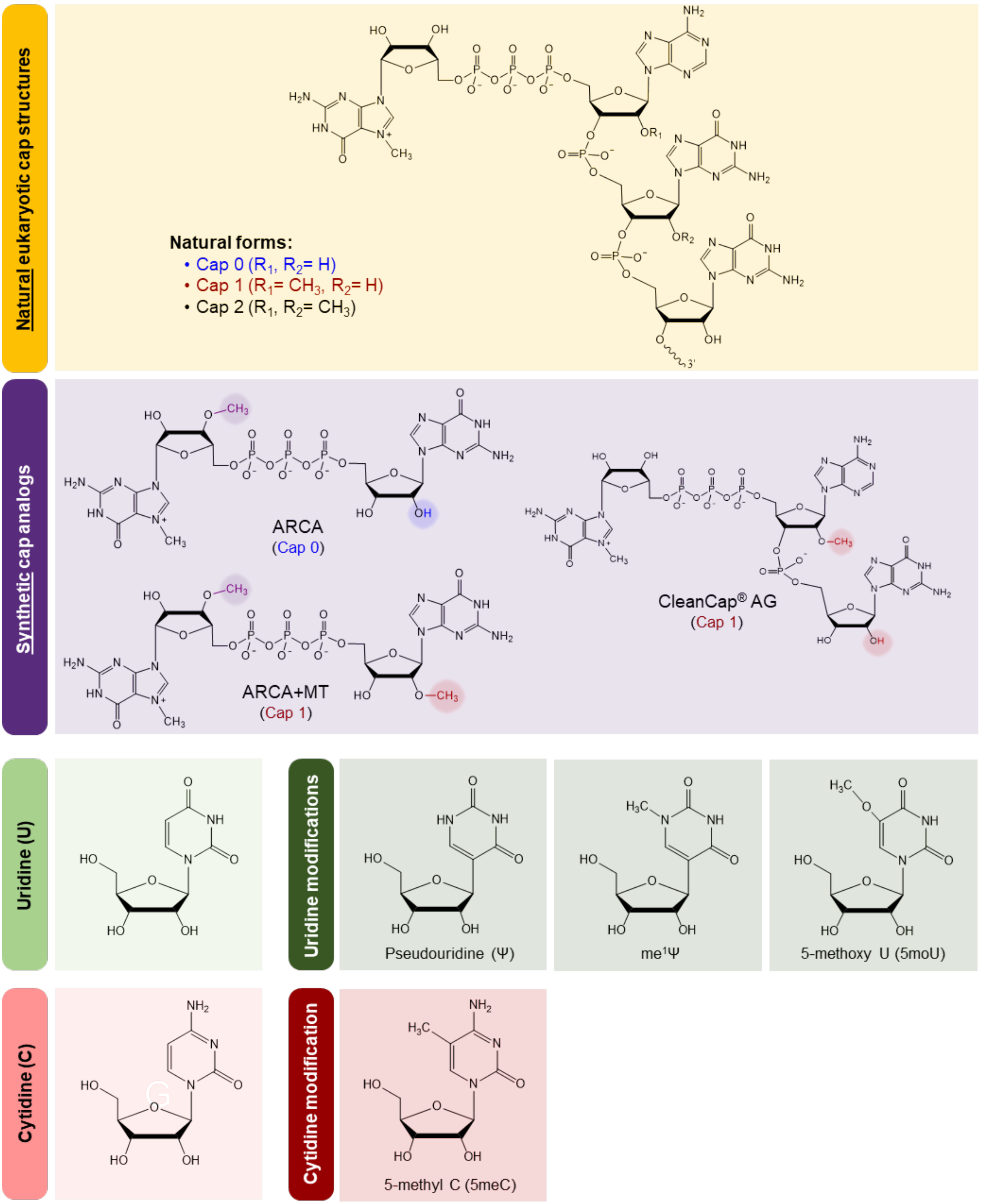

Figure S1. The upper panel (yellow box) depicts the chemical formula of naturally occurring eukaryotic cap structures in context of mRNA modification including Cap 0, Cap 1 and Cap 2 forms. Synthetic anti-reverse cap analogs used as initiator of *in vitro* mRNA synthesis process in form of dinucleotide ARCA, with and without extra methylation (Cap 0 vs. Cap 1), and trinucleotide CleanCap®AG, methylated at first adenosine (Cap1) (purple box). The changes of cap analogs compared to the natural cap are highlighted. The lower panels depict original and modified uridine and cytidine nucleoside implemented in this study; (ARCA: anti-reverse cap analogue, MT: methyl-transferase, Ψ: pseudouridine, me^{1}Ψ: N^1-Methylpseudouridine, 5moU: 5-methoxy-uridine, 5meC: 5-methyl-cytidine)

a

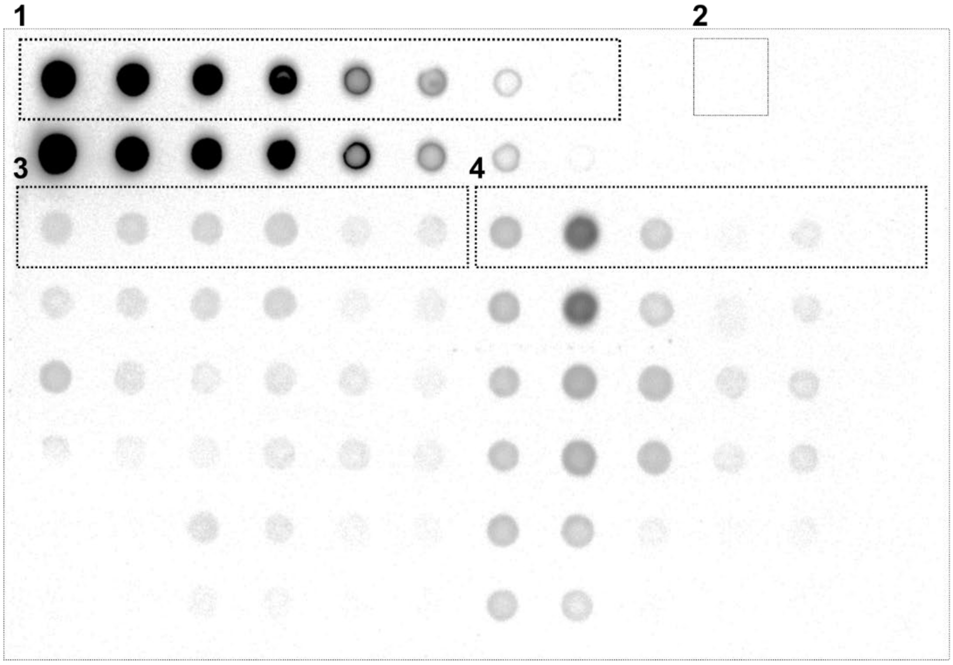

b

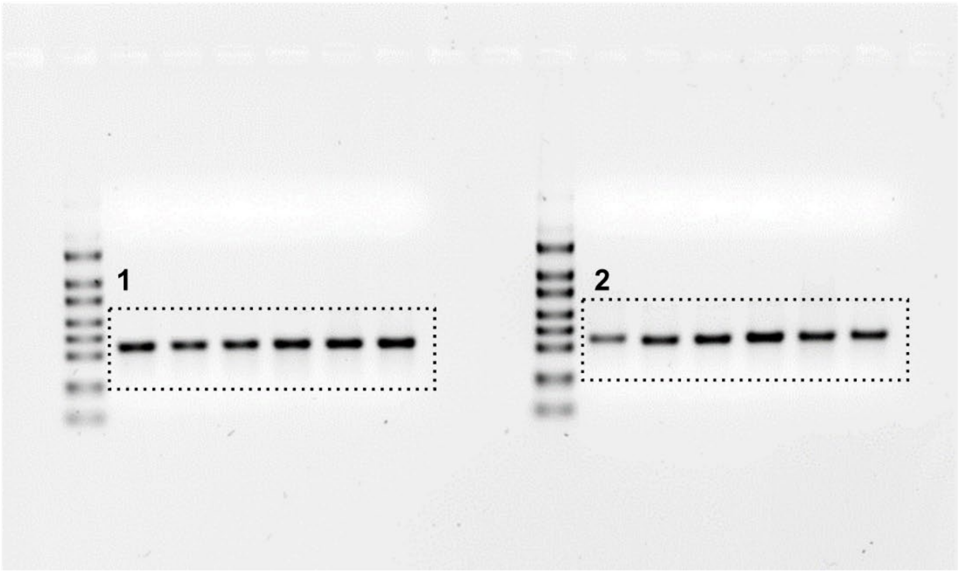

Figure S2. The uncropped image of **(a)** a dot blot membrane and **(b)** an agarose gel. **(a)** The dotted frames on dot blot correspond to samples as follows; 1) dsRNA standard series, 2) ssRNA as negative control, 3) cap modified IVT-mRNAs, 4) nucleotide modified IVT-mRNAs; all of which are presented in figure 3a-c. **(b)** The dotted frames on the agarose gel image correspond to 1) cap modified and 2) nucleotide modified IVT-mRNAs presented in figure 3b, c.

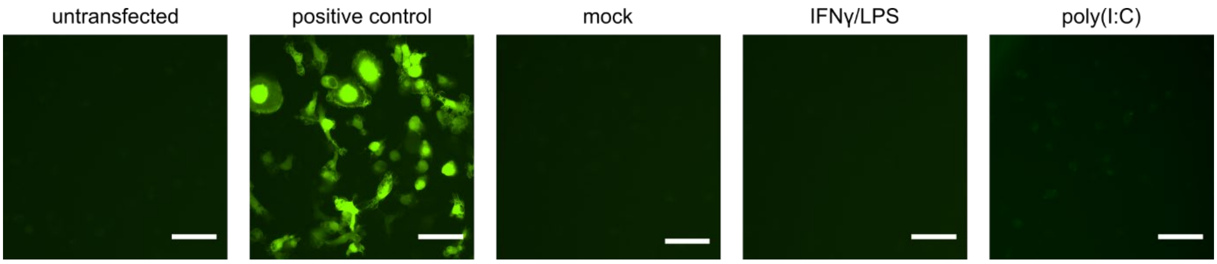

Figure S3. Fluorescent images of control samples, including untransfected, EGFP transfected positive control, mock transfected (LipoMM only), IFNγ/LPS treated as proinflammatory immune response positive control and poly(I:C) transfected macrophages as dsRNA-induced antiviral response positive control. Scale bar= 50 µm.

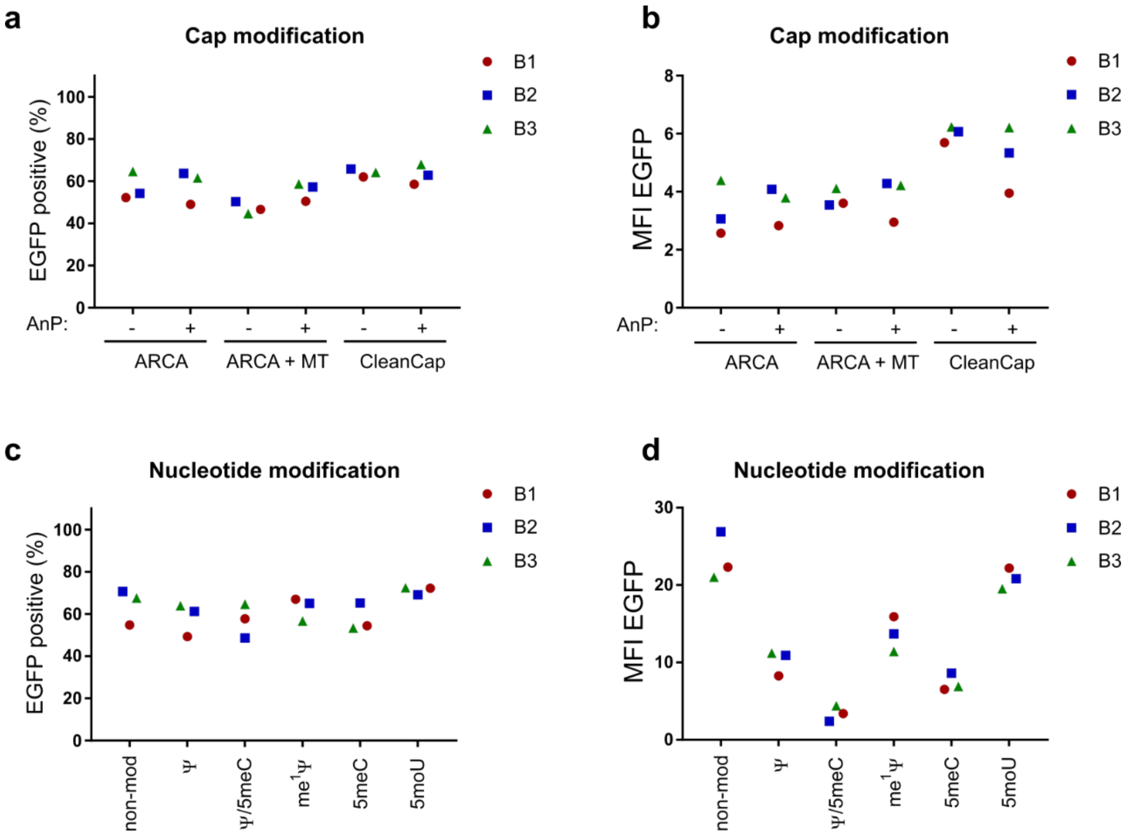

Figure S4. Evaluation of batch effects by comparison of three independently synthesized IVT-mRNA batches within the same donor; comparison of **(a)** transfection efficiency and **(b)** level of EGFP production between three batches of IVT-mRNA (B1-B3), with cap modification. Macrophages transfected IVT-mRNA with distinct nucleotide modification were evaluated in terms of **(c)** transfection efficiency and **(d)** level of EGFP production throughout three IVT-mRNA batches. (ARCA: anti-reverse cap analogue, MT:methyl-transferase, AnP: Antarctic phosphatase, Ψ: pseudouridine, me^{1}Ψ: N^1-Methylpseudouridine, 5moU: 5-methoxy-uridine, 5meC: 5-methyl-cytidine)

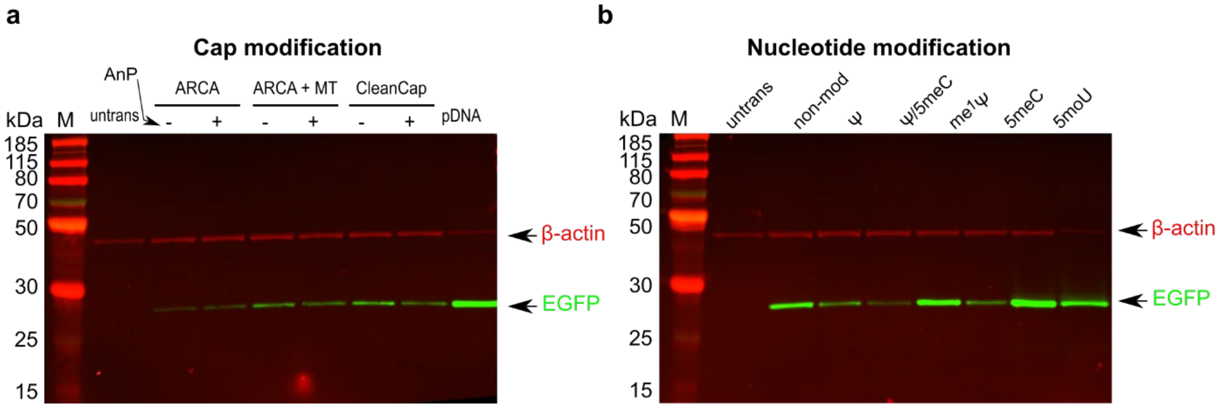

Figure S5. Protein expression evaluated by western blot analyses for macrophages transfected with either **(a)** cap modified or **(b)** nucleotide modified IVT-mRNA (ARCA: anti-reverse cap analogue, MT:methyl-transferase, AnP: Antarctic phosphatase, Ψ: pseudouridine, me^{1}Ψ: N^1-Methylpseudouridine, 5moU: 5-methoxy-uridine, 5meC: 5-methyl-cytidine)

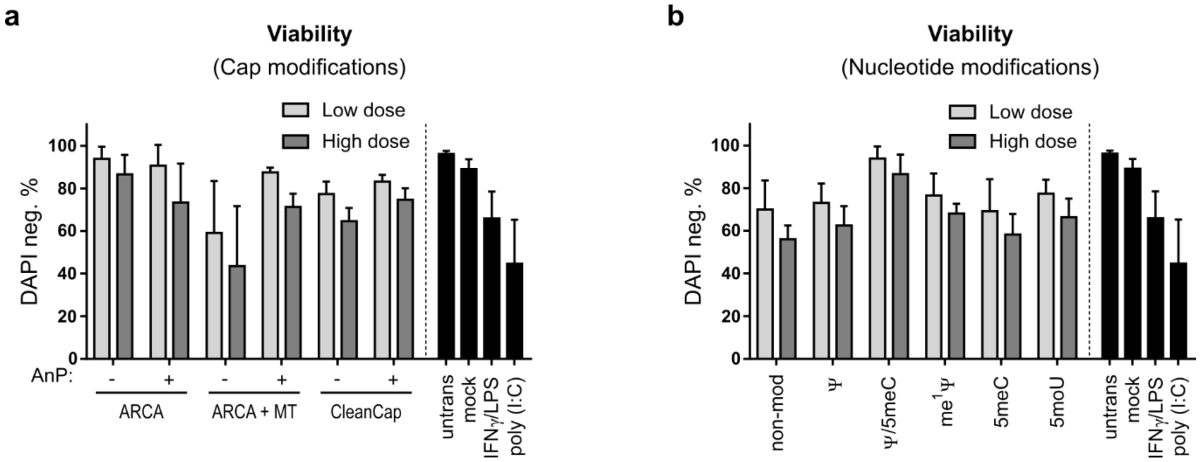

Figure S6. Viability of macrophages transfected with low dose and high dose of IVT-mRNA with chemical modification of either **(a)** cap structure or **(b)** nucleotides, evaluated by determining the percentage of DAPI-negative cells. Values are presented as mean ± SD, n = 3. (ARCA: anti-reverse cap analogue, MT: methyl-transferase, AnP: Antarctic phosphatase, Ψ: pseudouridine, me^{1}Ψ: N^1-Methylpseudouridine, 5moU: 5-methoxy-uridine, 5meC: 5-methyl-cytidine)

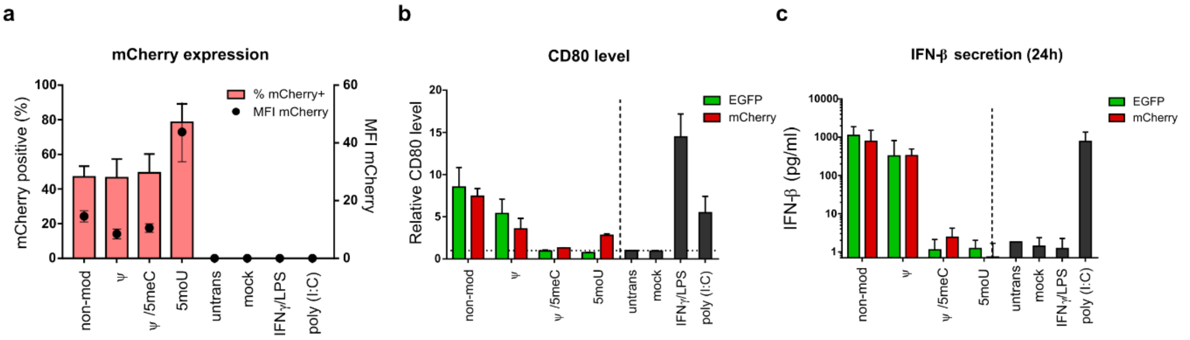

Figure S7. Macrophage response to transfection of mRNA coding for mCherry and EGFP with different nucleotide modification. (a) Transfection efficiency (bar graph, left axis), and mRNA expression level quantified by percentage of mCherry positive cells and mean fluorescent intensity of mCherry, respectively, (scattered dots, right axis), quantified using flow cytometry, 24 h post transfection. Comparison of CD80 level (b) and IFN-β secretion (c) by macrophages after IVT-mRNA transfection enconding for mCherry or EGFP with different nucleotide modifications, both measured 24 h after transfection. mCherry IVT-mRNA was transfected in low dose, i.e. 125 ng·mL^{-1}. Untransfected, poly(I:C)-treated, and LPS/IFN-γ-activated macrophages served as negative, positive and high control, respectively. Values are presented as mean ± SD. Error bars indicate SD of three independent experiments from three individual donors.

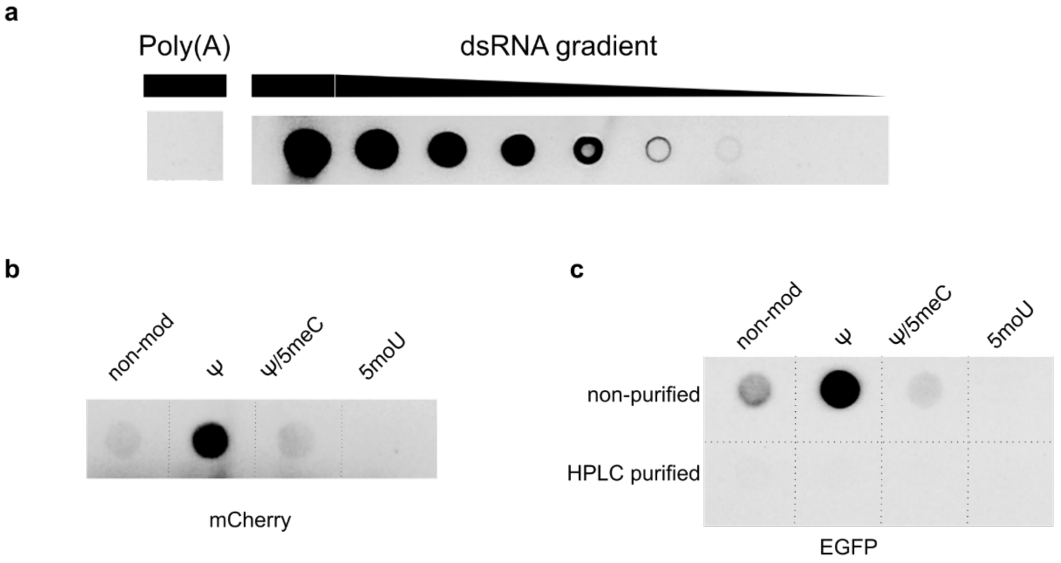

Figure S8. dsRNA detection by J2 antibody comparing non-purified with HPLC-purified IVT-mRNAs **(a)** Poly(A) as ssRNA negative control, and dsRNA positive control were blotted with the same amount as main samples (1000 ng/dot), dsRNA was 4-fold serial diluted (from left to right). **(b)** dsRNA content of mCherry encoding IVT-mRNA modified with different nucleotide modifications evaluated by J2 antibody binding. **(c)** Representative dot blots of EGFP encoding IVT-mRNAs with different nucleotide modifications comparing non-purified (top) versus HPLC-purified (bottom) samples. (Ψ: pseudouridine, 5moU: 5-methoxy-uridine, 5meC: 5-methyl-cytidine)

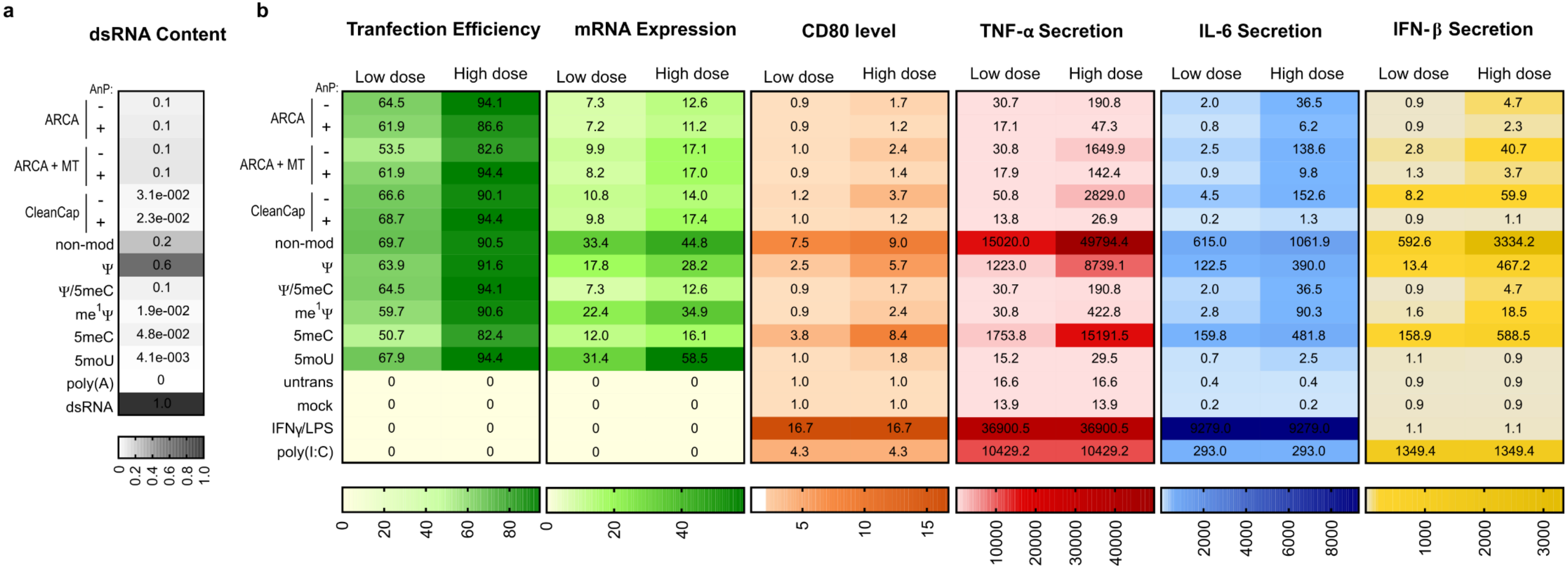

Figure S9. Overview of the properties and performance of IVT-mRNA with different cap and nucleotide modifications in terms of dsRNA content, protein expression, and inflammatory responses of transfected macrophages illustrated as heat map graphs. **(a)** dsRNA content, **(b)** (from left to right) transfection efficiency in percentage of EGFP positive cells, mRNA expression level measured by mean fluorescent intensity (MFI) of EGFP signal, and relative CD80 surface marker expression indicating the inflammatory responses, normalized MFI measured by flow cytometry. The cytokine secretion measured 24 h after transfection is presented as concentration (pg·mL^{-1}) of TNF-α, IL-6 and IFN-β. Untransfected, poly(I:C)-treated, and LPS/IFN γ-activated macrophages served as negative, positive and high control, respectively.

Table S1. Chemical modifications and process of preparation of IVT-mRNAs synthesized

	Sample ID	Chemical composition		Post-transcriptional modifications	
		Cap structure (type)	Nucleotides	2'-O-Methyltransferase treatment	Phosphatase treatment
Cap modified IVT-mRNA	ARCA/-	ARCA (Cap 0)	Ψ/5meC/A/G	-	-
	ARCA/+	ARCA (Cap 0)	Ψ/5meC/A/G	-	+
	ARCA+MT/-	ARCA (Cap 1)	Ψ/5meC/A/G	+	-
	ARCA+MT/+	ARCA (Cap 1)	Ψ/5meC/A/G	+	+
	CleanCap/-	CleanCap AG (Cap 1)	Ψ/5meC/A/G	-	-
	CleanCap/+	CleanCap AG (Cap 1)	Ψ/5meC/A/G	-	+
Nucleotide modified IVT-mRNA	non-mod	ARCA (Cap 0)	U/C/A/G	-	-
	Ψ	ARCA (Cap 0)	Ψ/C/A/G	-	-
	me^{1}Ψ	ARCA (Cap 0)	me^{1}Ψ/C/A/G	-	-
	5meC	ARCA (Cap 0)	U/5meC/A/G	-	-
	5moU	ARCA (Cap 0)	5moU/C/A/G	-	-
	Ψ/5meC (2nt. mod.)	ARCA (Cap 0)	Ψ/5meC/A/G	-	-

Sample ID refers to abbreviations which was used in text throughout. (ARCA: anti-reverse cap analogue, MT: methyl-transferase, Ψ: pseudouridine, me^{1}Ψ: N^1-Methylpseudouridine, 5moU: 5-methoxy-uridine, 5meC: 5-methyl-cytidine, 2nt. mod: two nucleotides were modified)
